# An investigation of the diagnostic, predictive, and prognostic impacts of three colonic biopsy grading systems for acute graft versus host disease

**DOI:** 10.1371/journal.pone.0256543

**Published:** 2021-08-26

**Authors:** Andreas Kreft, Katrin Hippe, Eva Maria Wagner-Drouet, Isabelle Ries, Arne Kandulski, Maike Büttner-Herold, Helmut Neumann, Daniela Weber, Ernst Holler, Mario Schindeldecker

**Affiliations:** 1 Institute of Pathology, University Medical Center Mainz, Mainz, Germany; 2 Institute of Pathology, University Hospital Regensburg, Regensburg, Germany; 3 3^rd^ Medical Department, Hematology, Oncology and Pneumology, University Medical Center Mainz, Mainz, Germany; 4 Department of Internal Medicine I, University Hospital Regensburg, Regensburg, Germany; 5 Department of Nephropathology, Institute of Pathology, Friedrich-Alexander-University Erlangen-Nürnberg (FAU) and University Hospital Erlangen, Erlangen, Germany; 6 1^st^ Medical Department, University Medical Center Mainz, Mainz, Germany; 7 Department of Internal Medicine III, University Hospital Regensburg, Regensburg, Germany; 8 Tissue Biobank, University Medical Center Mainz, Mainz, Germany; University of Kentucky, UNITED STATES

## Abstract

Acute graft versus host disease (aGvHD) is an important, life-threatening complication after allogeneic hematopoietic stem cell transplantation (alloHSCT). To investigate the value of multiple simultaneous colon biopsies in improving diagnostic accuracy in patients with aGvHD, we retrospectively analyzed 157 patients after alloHSCT. The biopsies were evaluated individually using three established histological grading systems (Lerner, Sale, and Melson). The maximum, minimum, median, and mean histological aGvHD grades were calculated for each patient, and the results were correlated with the Glucksberg grade of clinical manifestation of GvHD, steroid therapy status, and outcome. We found that multiple colon biopsies enhanced diagnostic sensitivity. Moreover, higher histological grades correlated with steroid therapy initiation and refractoriness; the latter particularly occurred when advanced damage was present in all samples and healthy colon mucosa was reduced or absent. On multivariate analysis, the minimal Lerner and Glucksberg grades for intestinal aGvHD were significantly associated with steroid treatment failure. Ninety-nine patients died. The median survival was 285 days after the biopsies were taken. Fifteen patients died from relapse of their underling disorder and 84 from other causes, mostly infection (53 patients) and GvHD (14 patients). Multivariate analysis revealed a significant association between none-relapse mortality and the mean Lerner grade, minimum Melson grade, Glucksberg organ stage, and platelet counts. Thus, we found the Lerner system to be superior to the other grading methods in most instances and histologic evaluation of multiple simultaneously obtained biopsies from the colon to result in a higher diagnostic yield, which helps plan systemic steroid treatment while predicting treatment response and outcome.

## Introduction

Intestinal acute graft versus host disease (aGvHD) is major cause of patient morbidity and mortality following allogenic hematopoietic stem cell transplantation (alloHSCT) performed for malignant and non-malignant hematologic disorders. The pathogenic mechanism is mediated by engrafted donor immune cells causing an allo-immunoreaction that leads to epithelial cell apoptosis, inflammation, and tissue injury [1]. AGvHD affects 20–30% of patients [2] and accounts for approximately 20% of deaths after alloHSCT [3]. Lower gastrointestinal aGvHD has the highest incidence [4] and is most associated with non-relapse mortality (NRM) and overall survival (OS) [5]. Typical symptoms are diarrhea as well as abdominal pain, ileus, bloody stool, and malabsorption in the more severe cases [5].

Thus, many investigators rely on colon biopsies for the histological diagnosis and grading of this condition [6–10]. However, there are two constraints using this approach. First, there are competing diagnostic and grading systems for colon aGvHD, with the Lerner [11], Sale [12], and Melson [13] methods being the most popular; to date, these have not been compared with respect to their diagnostic accuracy and prognostic impact. Second, previous analyses are generally based on a single biopsy per patient only [6, 7, 14]; this disregards the variations in aGvHD manifestations throughout the colon, which are profound [8–10, 15]. Thus, evaluating multiple biopsy sites per patient using the most appropriate diagnostic system would greatly enhance the histological assessment of aGvHD, thereby guiding both patient management as well as further scientific exploration.

Here, we investigated whether performing multiple simultaneous endoscopic colon biopsies from 157 patients who underwent alloHSCT improves diagnostic efficacy. Furthermore, histological findings were graded according to the Lerner, Sale, and Melson systems and compared to the clinical manifestations of aGvHD, as well as to the patients’ management and outcomes, to identify which of these grading methods is optimal.

## Materials and methods

### Patients

Using medical records recorded between 2006 and 2019 at the Institute of Pathology of University Medical Center Mainz and the Pathological Department of University Hospital Regensburg (both in Germany), we identified 157 patients (96 from Mainz and 61 from Regensburg) with sufficient biopsy tissue from at least three different sites on the colon acquired during the same endoscopic evaluation, which were performed 20–200 days after alloHSCT, and without clinical, histological, or immunohistological signs of infection or severe toxic drug effects. All these patients were included in our evaluation. Thus our collective was not further selected and was therefore considered to be representative for patients undergoing multiple biopsies of the colon after alloHSCT. The biopsies were taken as part of the clinical workup, they were not taken for propose of this study. The specimens were sent in separate containers for each anatomical site; only one biopsy series per patient was evaluated. Patient’s characteristics are given in Table 1.

**Table 1:** The patient’s characteristics.

Parameter	Value
Male/Female (n)	94 (60%) / 63 (40%)
Age (years)	
Median	58
Range	18–75
Underlying disease	
AML	79 (50%)
MDS	27 (17%)
CMPN	10 (6%)
ALL	9 (6%)
Lymphoma	20 (13%)
Myeloma	8 (5%)
Aplastic anemia	2 (1%)
Donor type	
Sibling HLA identical	27 (17%)
Unrelated HLA identical	89 (57%)
Unrelated HLA different	41 (26%)
Time after alloHSCT (days)	
Median	94
Range	20–197
Number of colon biopsies per series	
Median	5
Range	3–9

ALL, acute lymphocytic leukemia; alloHSCT, allogenic hematopoietic stem cell transplantation; AML, acute myeloid leukemia; CMPN, chronic myeloproliferatve neoplasm; HLA, human leukocyte antigen; MDS, myelodysplastic syndrome.

The patients underwent alloHSCT at the 3^rd^ Medical Department of the University Medical Center Mainz or the Department of Internal Medicine III at the University Hospital Regensburg. Lifelong follow-up was performed according to institutional guidelines; GvHD assessment was conducted according to clinical routine and clinical source data were reassessed according to the revised Glucksberg criteria [5]. Steroid treatment was initiated according to standard clinical procedures and standard response criteria were also used [16]. Endoscopies and biopsies were performed as clinicaly indicated. If cases showed affected colon mucosa it was the standart procedure to take biopsies only from affected colon mucosa and not from normal mucosa. Endoscopy reports were obtained from the clinical files and critically reviewed. When comparing them to histology, the endoscopic findings were categorized as normal (0), edema (1), colitis or erythema (2), erosion (3), and ulceration (4).

All data were completely de-identified for further evaluation.

### Histology

All colon biopsies included in this study were re-evaluated by one pathologist experienced in GvHD diagnostics (AK). For each Lerner grade [11, 17] ascribed per biopsy site using consensus criteria [18], the Sale [12] and Melson grades [13] were assigned based on a series of six to eight serial steps of hematoxylin and eosin staining for each sample (Table 2). Apoptotic crypt cell bodies were counted per 0.25 mm^2^.

**Table 2:** The histologic grading systems for intestinal acute graft-versus-host disease used in this study.

System	Grade 0	Grade 1	Grade 2	Grade 3	Grade 4
Lerner	Normal mucosa	Crypt cell apoptosis	Crypt destruction	Focal mucosa denudation	Diffuse mucosa denudation
Sale	Normal mucosa	Crypt abscess	Individual, non-contiguous crypt loss	Contiguous areas of two or more lost crypts	Denudation of the mucosa
Melson	Normal mucosa	Crypt cell apoptosis	Individual, non-contiguous crypt loss	Contiguous areas of two or more lost crypts	Complete crypt loss

## Statistics

The averages of independent groups that each consisted of one dependent scale variable and one explanatory nominal variable with two or more levels were compared using the Mann-Whitney U-test and Kruskal-Wallis test, respectively. Benjamini-Hochberg corrections were applied to reduce the effects of multiple testing and correct for the false discovery rate.

Fisher’s exact test was used for testing the null of independence of rows and columns in a contingency table with fixed marginals derived from two categorical variables. Spearman’s correlation was used to examine linear correlations between two numeric variables showing a non-normal distribution.

To evaluate the effects of histologic grades on steroid response, the data were fit to a generalized binomial linear regression model. Histological data, clinical parameters and scores were dichotomized utilizing R-code from a web-based software tool (cut-off finder), which optimizes the log-rank score, to identify the significant differences in survival outcomes that were associated with high versus low biomarker levels [19]. Univariate Cox regression analysis was performed to determine the associations between the patients’ basic characteristics or histological properties and their OS or NRM, respectively.

To account for the influence of established prognostic factors, hazard ratios (HRs) and 95% confidence intervals (CIs) were adjusted for histological properties using multivariate Cox proportional hazards regression analyses; stepwise variable selection was performed using the Akaike information criterion method via the R package MASS to determine significance. Kaplan-Meier plots, together with log-rank tests, were applied to identify characteristics or biomarkers that were associated with the patients’ OS or NRM.

To quantify intra-observer reproducibility and variations in histological scoring data within each individual biopsy series, kappa values applying linear weighting were calculated using the AgreeStat software (2015.6.1, Advanced Analytics, LLC, Gaithersburg, MD, USA). Upon re-evaluation of the 38 biopsies more than one year after their initial examination, intra-observer findings were excellently correlated according to the Fleiss’ kappa benchmark scale [20] with respect to the Lerner, Sale, and Melson grades (Cohen’s kappa: 0.89, 0.78, and 0.76 respectively). There were four, seven, and six differences, respectively, mostly concerning one grade.

### Ethics

This study was approved by the ethical committee of the medical board of Rhineland Palatinate (No. 0837.271.12 [8372]) and the ethical review board of the University of Regensburg (Nos. 17-618-101 and 09/059).

## Results

### Histologic diagnosis of aGvHD

**Fig 1:**
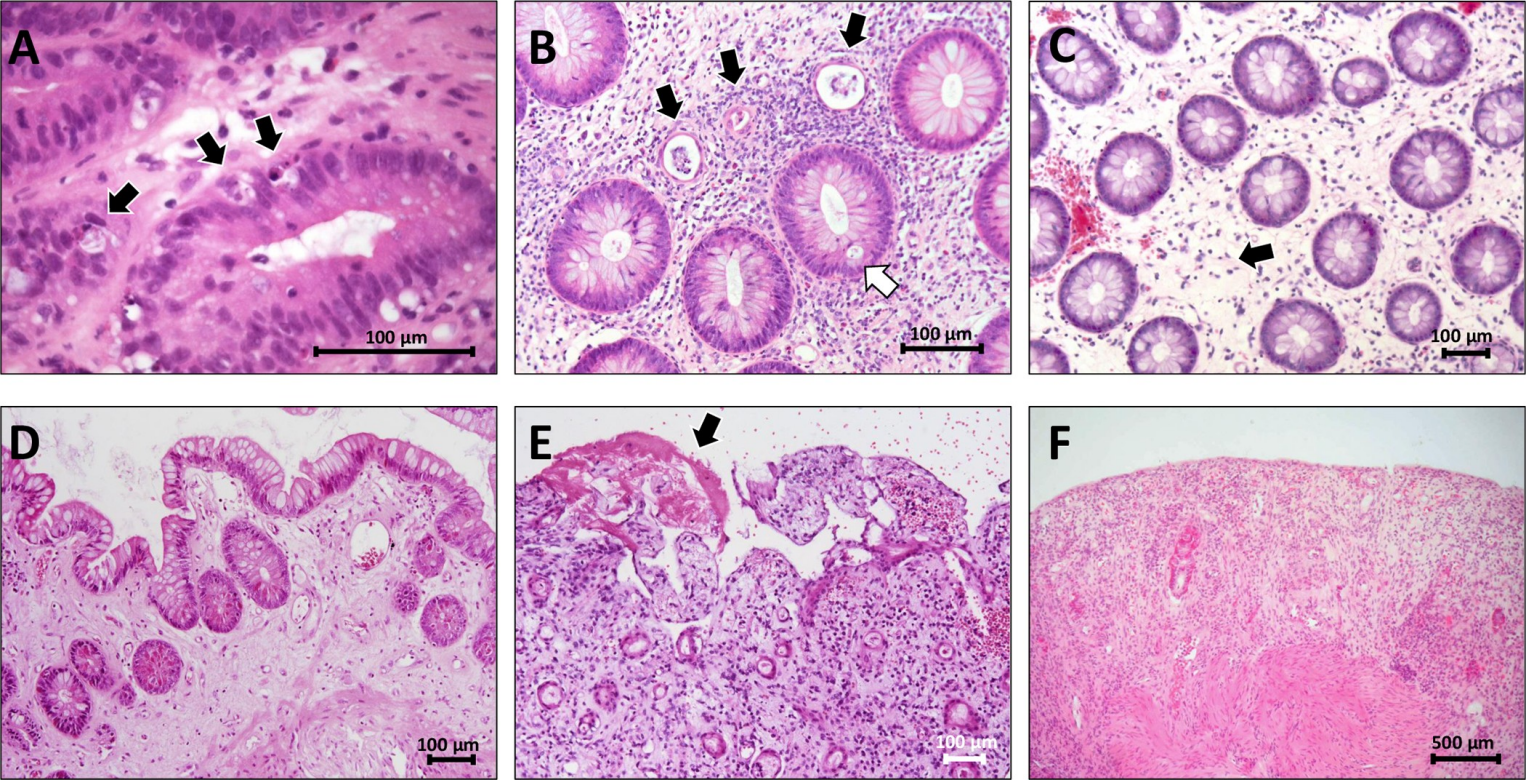
Histology of acute graft-versus-host disease of the colon. Crypt cell apoptosis: Apoptotic bodies consist of cell debris visible as dark particle in a lighter background (arrows) (Lerner grade 1, Sales grade 0, Melson grade 1. HE) (A). Crypt destruction: Degeneration of crypt epithelial cells, the lumen is filled with cell detritus and a few granulocytes (black arrows); a neighboring crypt exhibit an apoptotic body (white arrow) (Lerner grade 2, Sales grade 1, Melson grade 1. HE) (B). Individual crypt loss: Gap between preserved crypts the size of one crypt (arrow) (Sale grade 2, Melson grade 2), there is no crypt cell apoptosis or ongoing crypt destruction visible (therefore Lerner grade 0. HE) (C). Contiguous crypt loss: Wide gaps between residual crypts, Paneth cell metaplasia in the remaining crypts indicates chronic mucosa injury (Lerner grade 0 (because there is no ongoing destruction of crypt epithelial cells), Sales grade 3, Melson grade 3. HE) (D). Mucosa denudation: Loss of the superficial epithelial layer and fibrin deposit (arrow) (Lerner grade 3) and contiguous crypt loss (Sales grade 3, Melson grade 3. HE) (E). Diffuse mucosa denudation: Exhaustive mucosa destruction with near complete crypt loss (Lerner grade 4, Sales grade 4, Melson grade 4. HE) (F).

From our cohort of 157 patients, 749 biopsies were evaluated with a mean of five biopsies per patient (Table 1). The biopsies represented the entire histologic spectrum of colon aGvHD including normal mucosa, different numbers of apoptotic bodies, crypt destruction and loss, and mucosal denudation (Fig 1).

According to the Lerner classification, 150 patients revealed signs of aGvHD in at least one sample. The maximal histologic ratings for each series were grade 1 in 51 patients, grade 2 in 44, grade 3 in 26, and grade 4 in 29. Fifty patients had the same Lerner grades throughout all biopsy sites; among the remainder, the differences were one grade in 63 patients, two grades in 29, three grades in 14, and four grades in one. Thirty-eight patients had at least one biopsy with no apoptotic bodies or other signs of aGvHD, whereas other locations revealed histologic signs of aGvHD. The Fleiss kappa for the individual grading of the biopsies for each patient was 0.56.

The Sale classification, for which the threshold of aGvHD diagnosis is higher than that for the Lerner system, 139 patients showed grade ≥1 for aGvHD of the colon. A maximum of grade 1 was found in five patients, grade 2 in 28, grade 3 in 74, and grade 4 in 32. Forty-three patients had the same grade across all biopsies, whereas 36 series had differences in one grade, 31 in two grades, 40 in three grades, and seven in four grades. Seventy-four patients had at least one biopsy with grade 0 whereas the remaining biopsies were of grades ≥1. Here, the Fleiss kappa for individual grading in a series was 0.57.

Owing to the Melson classification’s inclusion of crypt loss as a sign of aGvHD, 152 patients were classified as having aGvHD grade ≥1. A maximum grade of 1 was found in 17 patients, grade 2 in 29, grade 3 in 74, and grade 4 in 32. Forty-five patients showed no differences in histologic grade between samples; in the remaining biopsy series, the differences were one grade in 55 patients, two grades in 42, three grades in 13, and four grades in two. Twenty-three patients had at least one biopsy with grade 0 whereas the other biopsies were of grades ≥1. The Fleiss kappa value for the biopsy series was 0.51.

**Fig 2:**
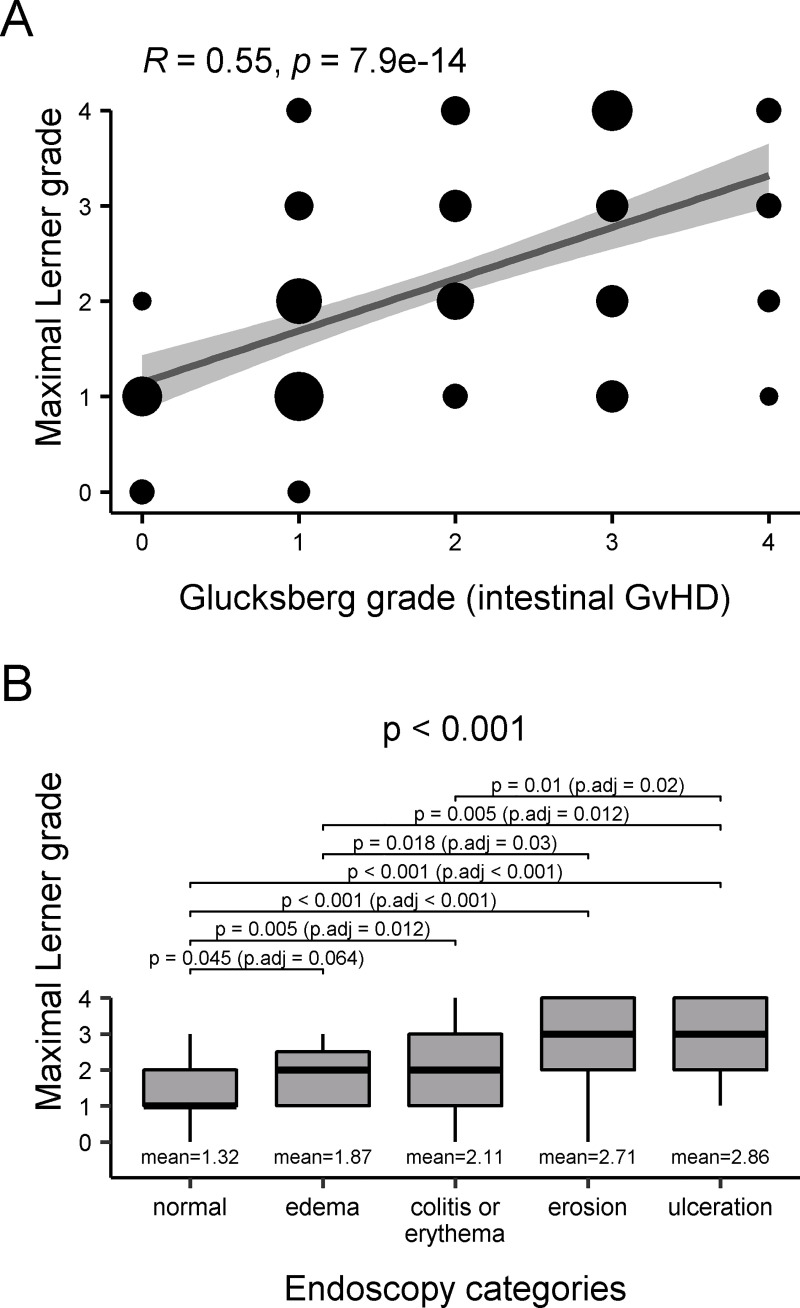
Correlation between the histological maximum Lerner grade of the samples obtained in the biopsy series with each of the Glucksberg grade of intestinal graft-versus-host disease (GvHD). Diameters of the dots correlate with number of cases linearly (A). Endoscopic findings in patients with acute GvHD (B).

The Glucksberg stage for intestinal GvHD was 0 in 19 patients, 1 in 57, 2 in 30, 3 in 39, and 4 in 12. The overall Glucksberg score was 0 in 13 patients, 1 in five, 2 and 3 in each of 60, and 4 in 19. There were 105, 22, and 11 patients with GvHD of the skin, liver, and oral mucosa, respectively. Correlation of all three histological grades with the Glucksberg stage of intestinal GvHD was highly significant in terms of the maximum, median, mean, and even minimum of all three grading system scores (each p ≤ 0.001) (S1–S3 Figs). However, the histological grading system found to be best correlated with the Glucksberg organ stage was the maximum Lerner grade, with an R-value of 0.55 (Fig 2A).

**Fig 3:**
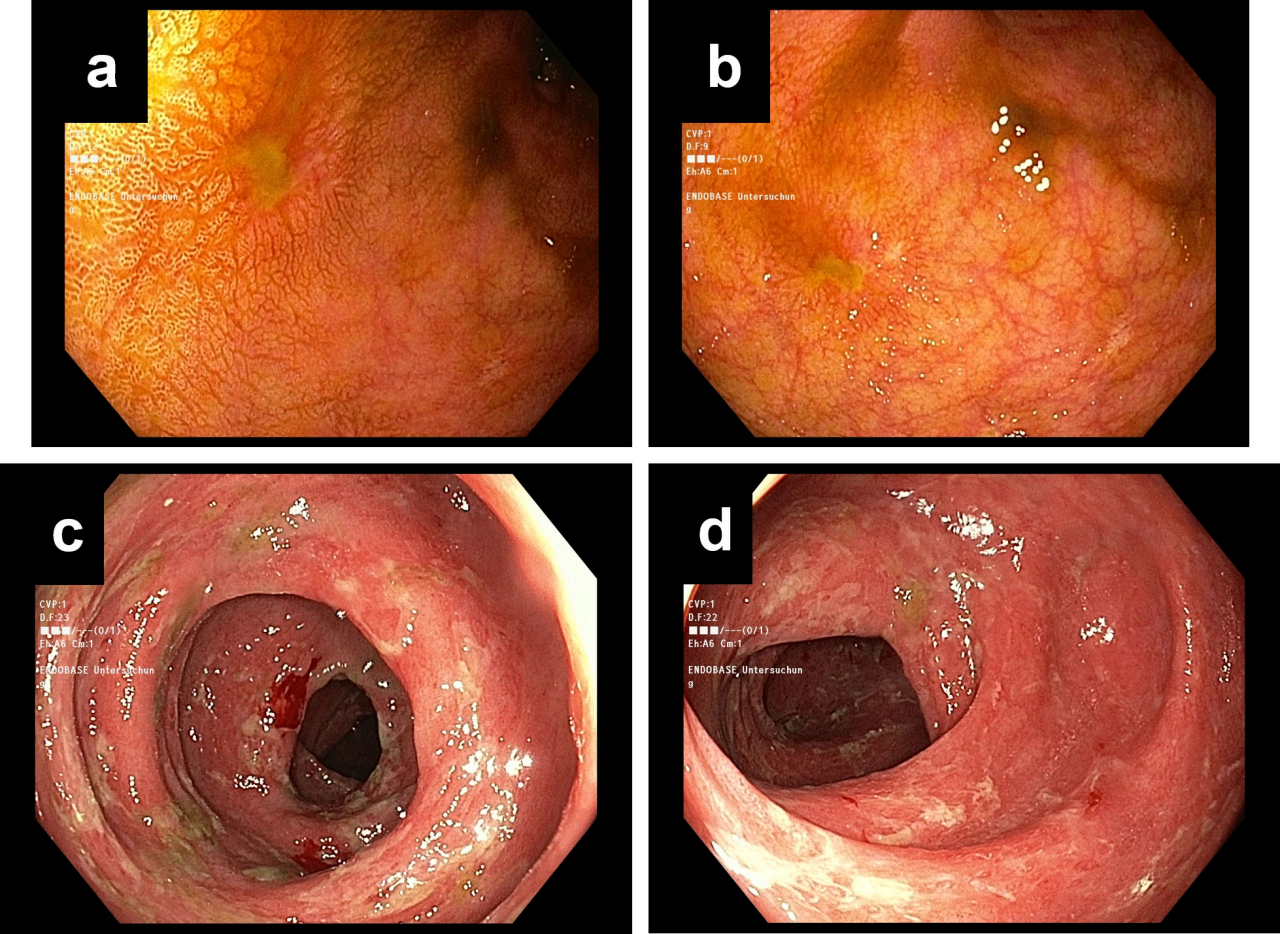
Endoscopic imaging of colonic manifestation of GvHD. Mild colitis with small erosions and very mild localized edema (A, B). Severe changes with generalized, pronounced edema, fibrinous exudation and multifocal granular ulceration (C, D).

Endoscopic data were available for 149 patients and based on the categories ‘normal’, ‘edema’, ‘erythema/colitis’, ‘erosion’ and ‘ulceration’, endoscopy mirrored all histological grades including the maximum, median, mean, and minimum, as well as the Glucksberg organ stage of intestinal aGvHD (all p ≤ 0.001) (Figs 2B and 3). However, of 40 patients with normal endoscopic findings, 27 showed crypt loss histomorphologically, 20 had focally increased apoptotic bodies, 12 had crypt destruction, and three had mucosal erosion.

### Prediction

Data on steroid therapy were available for 151 patients, of whom 128 received systemic steroid therapy; 90 patients in this group responded whereas 38 did not. Of the 23 patients who did not receive steroid therapy, two were steroid intolerant. Patients, which were refractory to steroids received a second line therapy including tacrolimus, etanercept, antithymocyte globulin, and extracorporeal photopheresis.

**Fig 4:**
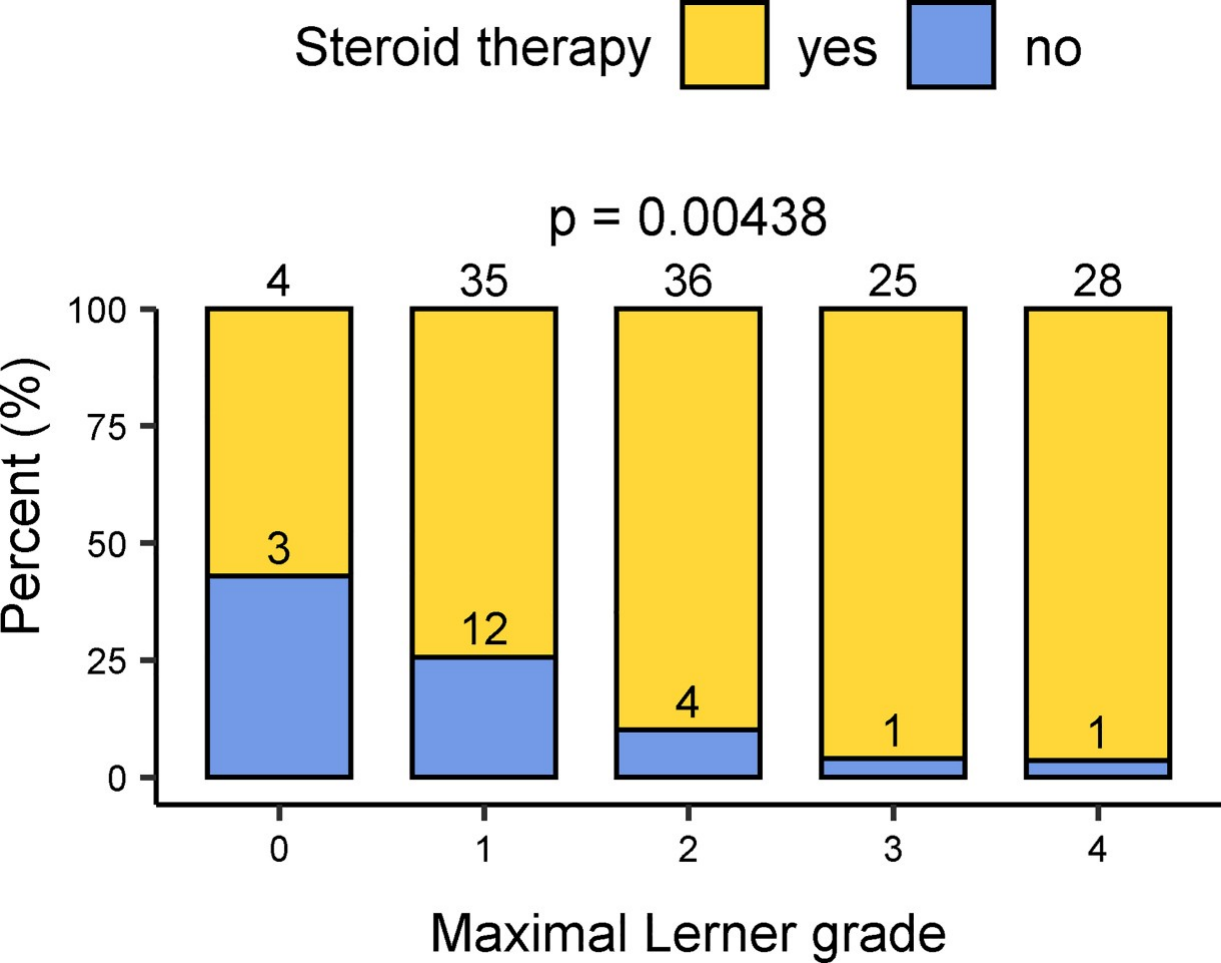
Comparison between the application of steroid therapy and the maximum histologic Lerner grade of a biopsy series. Numbers in bar charts indicate the count of cases for each group.

Patients with higher histological grades were more likely to receive steroid therapy. The correlation was most significant for the maximum Lerner grade (p ≤ 0.01) (Fig 4), the mean Lerner and Sale grades, and the maximum and mean Melson grades (each p ≤ 0.05) (S4–S6 Figs). Although the count of apoptotic bodies significantly correlated with the application of steroid treatment, there was no apparent threshold value delineating patients with or without therapy (S7 Fig).

**Fig 5:**
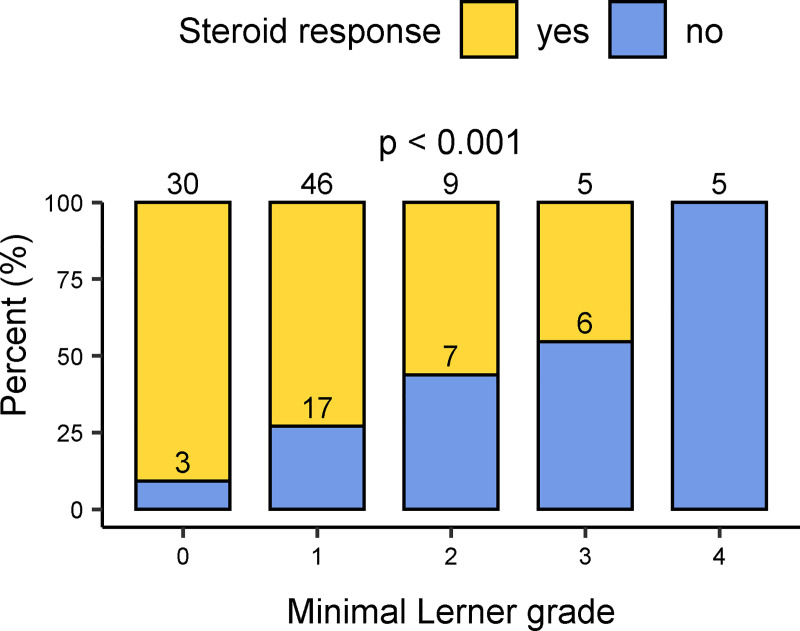
Comparison between the response to steroid therapy and the minimum histologic Lerner grade of a biopsy series. Numbers in bar charts indicate the count of cases for each group (only patients having received steroid therapy are included (n = 128)).

We found that patients with higher histological grades had inferior responses to steroid therapy. This was significant when using any of the three scoring systems and (in contrast to the application of steroids) was more clearly associated with the minimum and median biopsy series values (Fig 5 and S8–S10 Figs). Sensitivity to steroid therapy was also significantly associated with the Glucksberg organ stage of intestinal GvHD and with the overall Glucksberg score; in both, higher scores were less favorable (each p < 0.001).

On multivariate generalized linear regression analysis, the Glucksberg grade of intestinal aGvHD retained its highly significant association with the sensitivity to steroids (p < 0.001). With respect to histologic grades, only the minimum Lerner grade remained significant (p = 0.01) (S11 Fig).

### Outcome

Ninety-nine of the 157 patients (63%) died during the observation period, most within the first year after the biopsy (median 285 days; range 51–2 366 days). The causes of death were infection (n = 53), relapse of the underlying disease (n = 15), GvHD (n = 14), cardiovascular events (n = 9), solid tumors (n = 3), renal disease (n = 2), leukoencephalopathy (n = 2), and post-transplant lymphoproliferative disease (n = 1). The median observation time for the surviving patients was 1 439.5 days (range 460–4 647 days).

**Fig 6:**
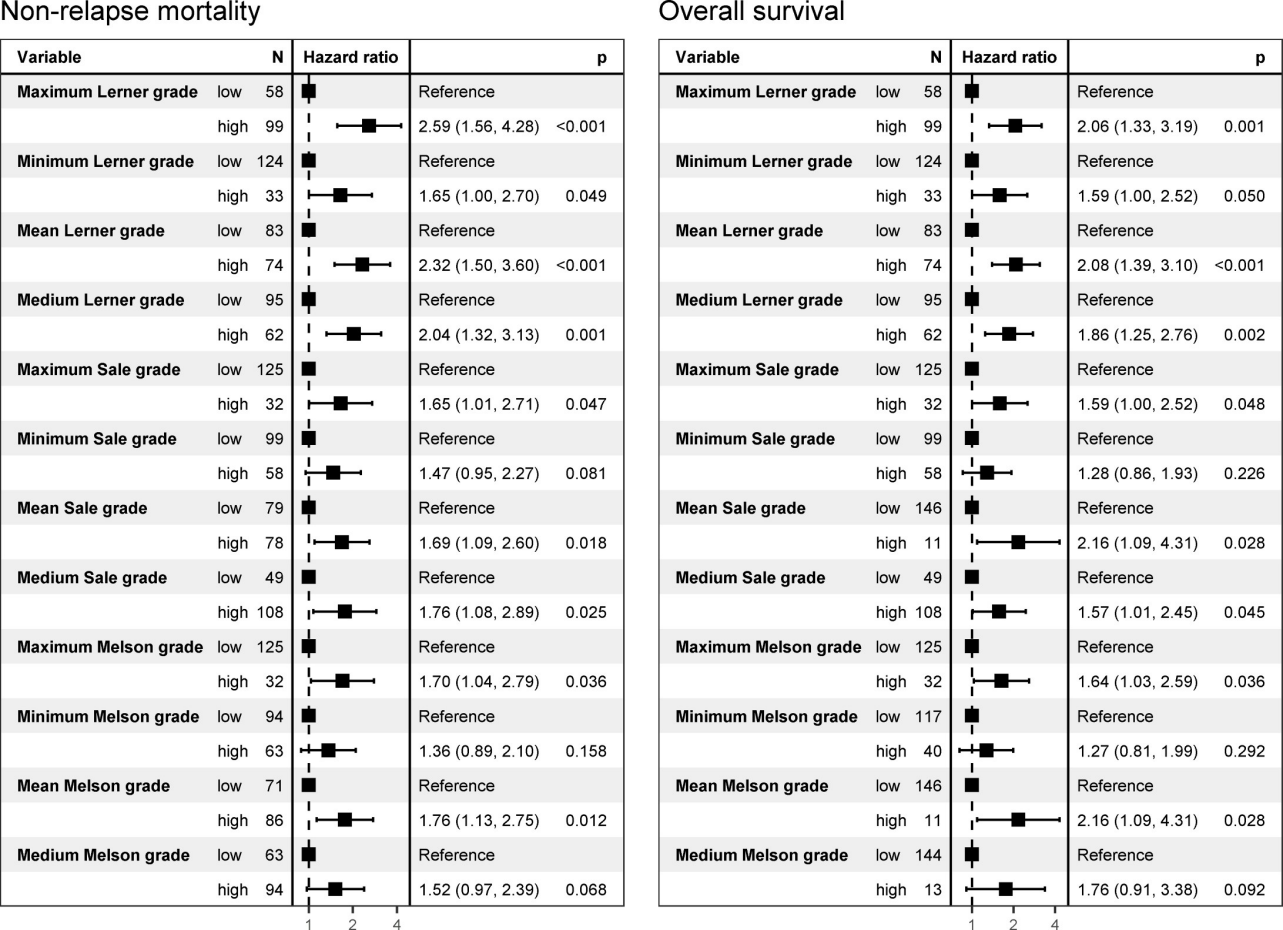
Univariate Cox regression analysis of non-relapse mortality and overall survival on histological grades.

After univariate survival analysis, the Lerner system was found to be the histological grading system best associated with OS and NRM, with highly significant results when considering the maximum and mean grades of the biopsy series. The maximum and mean values obtained using the other grading systems were also associated with NRM, but not as strongly as the Lerner grade (Fig 6). The cut-off between groups with good versus poor prognoses was found to be between grades 1 and 2 for most evaluations; those for the Sale and Melson maximums were between grades 3 and 4, and that for the Sale median was between grades 0 and 1.

Those patients, who had more than three colon biopsies, revealed a tendency for a shorter OS and a higher NRM (S14 Fig).

NRM and OS among patients who were not administered steroids (excluding those who were steroid-intolerant) was almost equal to that of steroid responders (p = 0.64, HR = 1.20, CI = 0.56–2.57, p = 0.56, HR = 1.23, CI = 0.62–2.41 respectively), and was significantly better than that in steroid-refractory patients (p = 0.01, HR = 2.70, CI = 1.23–5.92, p = 0.02, HR = 2.32, CI = 1.14–4.47 respectively) (S12 Fig).

**Fig 7:**
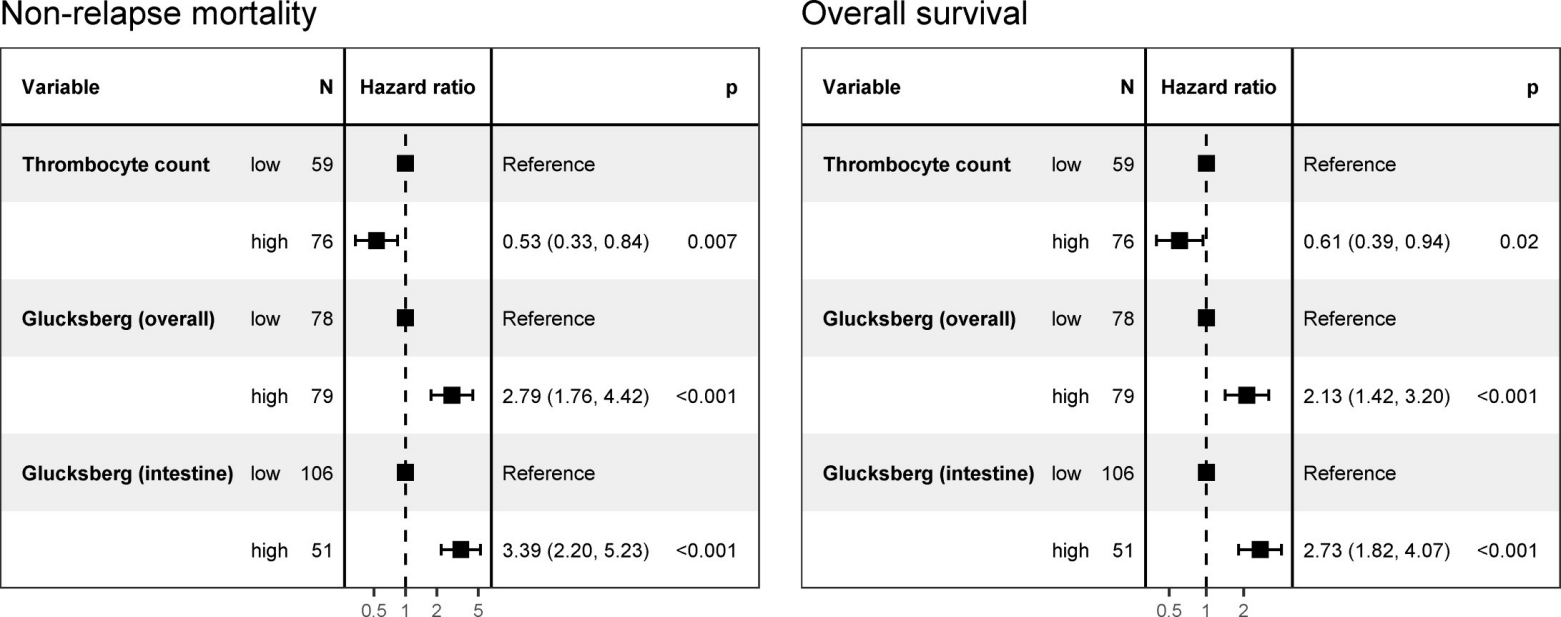
Univariate Cox regression analysis of non-relapse mortality and overall survival on thrombocyte count, Glucksberg grade of the graft-versus-host disease overall, and Glucksberg grade of the intestine.

A higher Glucksberg grade value for intestinal GvHD as well as the overall Glucksberg score were both strongly associated with unfavorable NRM and OS (each p ≤ 0.001) (Fig 7). Platelet count data were available from 135 patients; a low platelet-count (i.e., <70 500/μL) was significantly associated with poorer NRM (p = 0.007) and OS (p = 0.02).

**Fig 8:**
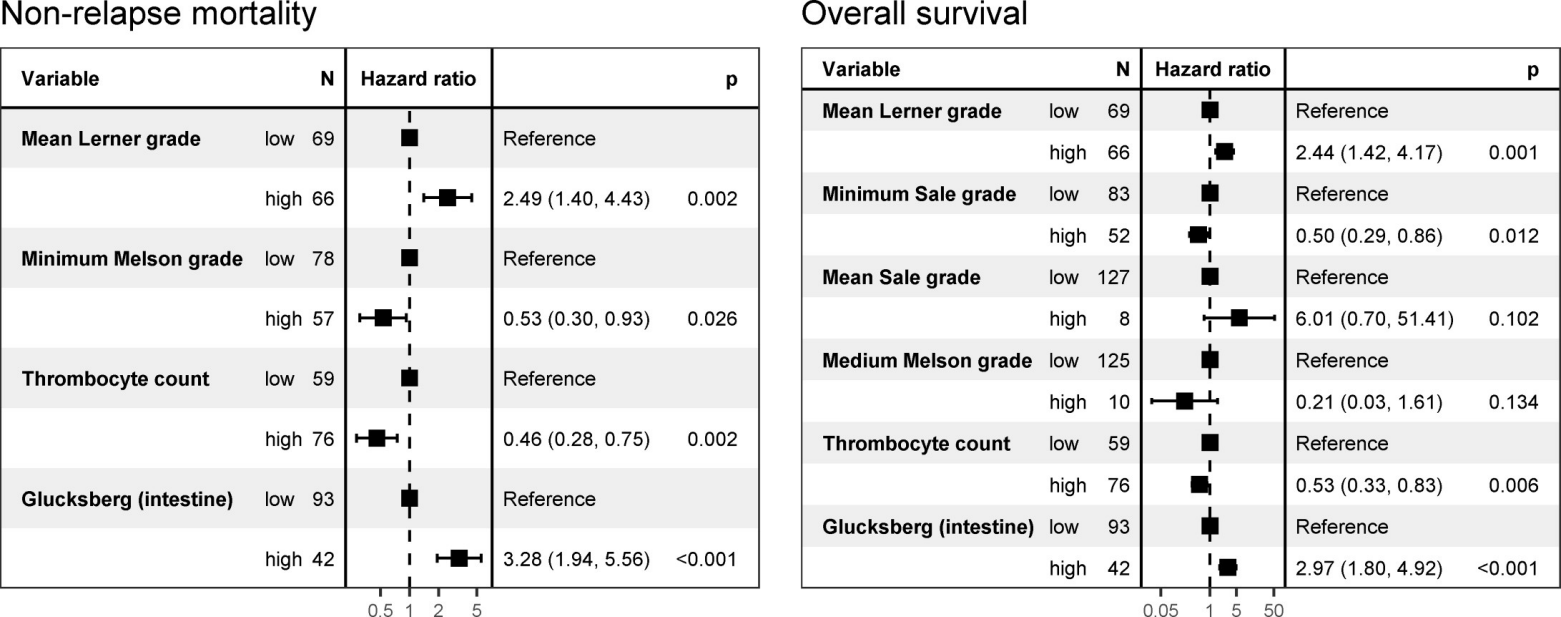
Multivariate Cox regression analysis of non-relapse mortality and overall survival on covariates selected using the Akaike information criterion backward stepwise variable method.

After multivariate survival analysis of the impact of the histological grades only the mean Lerner grade was significantly associated with OS and NRM. The minimum Sale grade was only associated with the OS and the minimum Melson grade for OS after performing Akaike information criterion backwards selection from 17 initial covariates (Fig 8). The platelet count and Glucksberg grade for intestinal GvHD were statistically significant for both the OR and the NRM.

## Discussion

We investigated the impact of an extensive examination of the colon’s histopathology on the accurate diagnosis of aGvHD. By obtaining multiple biopsies and comparing different scoring systems, we produced a markedly more representative and diverse database than those of previous histologic studies. This approach focuses on the substantial variations in the manifestations of colonic aGvHD in a single patient as well as the different histologic scoring systems applied. To further address the clinical validity of our results, we correlated our histologic findings with the diagnoses and outcomes of patients.

First, we confirmed the striking variation in the histologic manifestations of aGvHD within the colons of individual patients [8–10, 15]. The histological diagnosis of GvHD would have been missed in 20–50% of the patients in our cohort (depending on the diagnostic criteria used) if only one biopsy had been acquired and evaluated. Moreover, there was prominent variability in histological scores between the three grading methods.

To address the clinical implications of the various histological findings, we analyzed the correlations between the different aGvHD grades and whether the patients were administered steroids. We found that higher maximum histological grades under any system correlated strongest with steroid therapy. However, we could not establish a histological cut-off to differentiate clinically insignificant aGvHD from significant counterparts by quantifying apoptotic bodies [14, 21], even though the apoptotic body counts correlated with the application of steroid therapy. An increase in apoptotic bodies was observed in patients without clinically apparent intestinal aGvHD who had macroscopically bland mucosa; this is consistent with our previously published data [22]. Moreover, patients with histologically more advanced aGvHD who exhibited crypt destruction or mucosal loss were not automatically treated with steroids; thus, our results reinforced the concept of a gray zone in terms of the histologic diagnosis of significant aGvHD [7, 23].

We also found that patients with higher histological aGvHD grades in any of the grading systems were less responsive to steroid therapy, as were those with higher Glucksberg grades of intestinal GvHD; this was consistent with previous studies [13, 24]. However, we found that sensitivity to steroids was better correlated with the minimal histologic grades rather than with the maximal. Higher minimal grades indicate extensive mucosal damage with little or no preserved mucosa, whereas lower minimal grades indicate that the remaining mucosa is of better quality. Well-maintained mucosa may have better regenerative capacity compared to the situation when mucosal destruction is more severe, given that extensive proliferation (which occurs subsequent to serious mucosal damage in aGvHD) may exhaust the regenerative capacity of intestinal stem cells [25].

Previous studies found that a mere increase in apoptotic bodies [26] and/or histologic grade 1 aGvHD [6] are correlated with the prognosis. However, the NRM among our patients with histologic grade 1 aGvHD did not differ from that in patients without histologic aGvHD according to almost all evaluations. We found that the cut-off value for predicting which patients are at a higher risk of NRM was between histological grades 1 and 2 as per most univariate and two multivariate analyses. Thus, not only are grades 3 and 4 poor prognostic factors as reported by other groups [7, 27], but also grade 2, which is defined by crypt destruction or loss, indicating that the damage to the mucosal architecture may have reached deep into the stem cell zones, thereby weakening regeneration potential.

We found that all three histological grading methods offered useful information. However, we noticed that the Lerner system was best correlated with clinical data. This grading system focuses on active GvHD by including apoptosis, crypt destruction, and mucosal denudation; these features provide good histologic evidence of an active disease that is treatable by immunosuppressive therapy. The shortcoming of this system is that it neglects structural chronic damage to the mucosa, whereas the Melson and Sale systems include it as crypt loss. Thus, along with the mean Lerner grade, we found that the Melson minimum grade, indicating the amount of preserved mucosa, is an independent predictor of survival on multivariate analysis.

Regarding the subgroup of AML patients, who comprise almost the half of our collective, we were able to reproduce the significant correlation of the Glucksberg score of intestinal GvHD, the histologic scores, the correlation of the histological scores as well as the endoscopic findings. The association of histologic grades and steroid therapy was less strong but the significant correlation of the histological grade and the response to steroid therapy was maintained. Upon univariate analysis of OS and NRM the thrombocyte count and some histological scores lost their prognostic impact. However, in multivariate analysis the overall Glucksberg score and some histological scores gained significance (S15–S34 Figs). Thus, our results were essentially reproducible in the subgroup of AML patients. This may show that the impact of histology in aGvHD diagnostic is not altered by the underlying disease and could confirm our results.

The retrospective design and the evaluation of patients with multiple biopsies only may have introduced a bias towards more severely ill patients in our study since most of the patients after alloHSCT got no endoscopy or only single biopsies. This overrepresentation of the more severely ill patients may have an impact on the calculation of the diagnostic sensitivity, prediction, and prognosis. However, we could include several patients without or with only limited GvHD signs, which may at least partly compensate the bias. Another limitation of our study may be that we could not give specific localizations with the highest diagnostic yield, since we were not given this information in a sufficient number of patients. However, we previously demonstrated that the right colon is the most heavily involved site of the large intestine in patients with aGvHD [15]. Taking multiple biopsies can possibly consist of taking tissue samples from normal appearing mucosa along with those from areas pertaining active disease, possibly spoiling the diagnostic benefit of multiple biopsies. This cannot be monitored in a retrospective study. However, the standards of both endoscopy departments contributing to our study do not include this practice. Too, patients with more than three colon biopsies did have a tendency for a poorer survival. Thus, it is rather unlikely that taking normal biopsies in addition to specimens from affected mucosa has a relevant impact on our evaluation.

In conclusion, we demonstrated that the histologic evaluation of multiple biopsies obtained from the colon in a single endoscopic session is optimal for diagnosing aGvHD histologically. Moreover, the application of the Lerner system produces the best diagnostic information compared to other grading methods. Applying this diagnostic approach can improve decision-making in terms of steroid treatment as well as the prediction of patients’ responses and NRM and may therefore be recommended for the endoscopic and histological workup in patients with aGvHD.

## Supporting information

S1 Data (XLSX)

S1 Fig Linear correlation of Lerner grades and Glucksberg (intestinal GvHD). Diameters of dots correspond to case numbers linearly. Light grey bands symbolize 95% confidence intervals and dark grey lines linear regression lines.
(TIF)

S2 Fig Linear correlation of sale grades and Glucksberg (intestinal GvHD). Diameters of dots correspond to case numbers linearly. Light grey bands symbolize 95% confidence intervals and dark grey lines linear regression lines.
(TIF)

S3 Fig Linear correlation of Melson grades and Glucksberg (intestinal GvHD). Diameters of dots correspond to case numbers linearly. Light grey bands symbolize 95% confidence intervals and dark grey lines linear regression lines.
(TIF)

S4 Fig Comparison of steroid therapy and Lerner grades. Graphical illustrations of contingency tables displaying case numbers and overall p-values of fisher´s exact count test. Mean and median grades are categorized in ranges.
(TIF)

S5 Fig Comparison of steroid therapy and sale grades. Graphical illustrations of contingency tables displaying case numbers and overall p-values of fisher´s exact count test. Mean and median grades are categorized in ranges.
(TIF)

S6 Fig Comparison of steroid therapy and Melson grades. Graphical illustrations of contingency tables displaying case numbers and overall p-values of fisher´s exact count test. Mean and median grades are categorized in ranges.
(TIF)

S7 Fig Comparison of steroid therapy and apoptotic bodies. Box plots showing differences between amount of apoptotic bodies and steroid therapy. Displayed p-values derived from unpaired Mann-Whitney U-test.
(TIF)

S8 Fig Comparison of steroid response and Lerner grades. Graphical illustrations of contingency tables displaying case numbers and overall p-values of fisher´s exact count test. Mean and median grades are categorized in ranges.
(TIF)

S9 Fig Comparison of steroid response and sale grades. Graphical illustrations of contingency tables displaying case numbers and overall p-values of fisher´s exact count test. Mean and median grades are categorized in ranges.
(TIF)

S10 Fig Comparison of steroid response and Melson grades. Graphical illustrations of contingency tables displaying case numbers and overall p-values of fisher´s exact count test. Mean and median grades are categorized in ranges.
(TIF)

S11 Fig Multivariate generalized linear model of steroid response versus Glucksberg (intestinal GvHD) and Lerner grades. Forest plot of regression result depicting case numbers, odds ratios, confidence intervals and p-values.
(TIF)

S12 Fig Univariate COX regression of non-relapse mortality versus steroid application and response. Forest plot of regression result depicting case numbers, hazard ratios, confidence intervals and p-values.
(TIF)

S13 Fig Univariate COX regression of overall survival versus steroid application and response. Forest plot of regression result depicting case numbers, hazard ratios, confidence intervals and p-values.
(TIF)

S14 Fig Univariate COX regression of overall survival and non-relapse mortality versus biopsy count. Forest plot of regression result depicting case numbers, hazard ratios, confidence intervals and p-values.
(TIF)

S15 Fig Correlation between the histological maximum Lerner grade of the samples obtained in the biopsy series with each of the Glucksberg grade of intestinal graft-versus-host disease (GvHD) in patients with AML disease. Diameters of dots correlate with number of cases linearly (A). Endoscopic findings in patients with acute GvHD (B).
(TIF)

S16 Fig Comparison between the application of steroid therapy and the maximum histologic Lerner grade of a biopsy series in patients with AML disease. Graphical illustration of contingency table displaying case numbers and overall p-value of fisher´s exact count test.
(TIF)

S17 Fig Comparison between the response to steroid therapy and the minimum histologic Lerner grade of a biopsy series in patients with AML disease. Graphical illustration of contingency table displaying case numbers and overall p-value of fisher´s exact count test.
(TIF)

S18 Fig Univariate Cox regression analysis of non-relapse mortality and overall survival on histological grades in patients with AML disease. Forest plots of regression results depicting case numbers, hazard ratios, confidence intervals and p-values.
(TIF)

S19 Fig Univariate Cox regression analysis of non-relapse mortality and overall survival on thrombocyte count, Glucksberg grade of the graft-versus-host disease overall, and Glucksberg grade of the intestine in patients with AML disease. Forest plots of regression results depicting case numbers, hazard ratios, confidence intervals and p-values.
(TIF)

S20 Fig Multivariate Cox regression analysis of non-relapse mortality and overall survival on covariates selected using the Akaike information criterion backward stepwise variable method in patients with AML disease. Forest plots of regression results depicting case numbers, hazard ratios, confidence intervals and p-values.
(TIF)

S21 Fig Linear correlation of Lerner grades and Glucksberg (intestinal GvHD) in patients with AML disease. Diameters of dots correspond to case numbers linearly. Light grey bands symbolize 95% confidence intervals and dark grey lines linear regression lines.
(TIF)

S22 Fig Linear correlation of Sale grades and Glucksberg (intestinal GvHD) in patients with AML disease. Diameters of dots correspond to case numbers linearly. Light grey bands symbolize 95% confidence intervals and dark grey lines linear regression lines.
(TIF)

S23 Fig Linear correlation of Melson grades and Glucksberg (intestinal GvHD) in patients with AML disease. Diameters of dots correspond to case numbers linearly. Light grey bands symbolize 95% confidence intervals and dark grey lines linear regression lines.
(TIF)

S24 Fig Comparison of steroid therapy and Lerner grades in patients with AML disease. Graphical illustrations of contingency tables displaying case numbers and overall p-values of fisher´s exact count test. Mean and median grades are categorized in ranges.
(TIF)

S25 Fig Comparison of steroid therapy and sale grades in patients with AML disease. Graphical illustrations of contingency tables displaying case numbers and overall p-values of fisher´s exact count test. Mean and median grades are categorized in ranges.
(TIF)

S26 Fig Comparison of steroid therapy and Melson grades in patients with AML disease. Graphical illustrations of contingency tables displaying case numbers and overall p-values of fisher´s exact count test. Mean and median grades are categorized in ranges.
(TIF)

S27 Fig Comparison of steroid therapy and apoptotic bodies in patients with AML disease. Box plots showing differences between amount of apoptotic bodies and steroid therapy. Displayed p-values derived from unpaired Mann-Whitney U-test.
(TIF)

S28 Fig Comparison of steroid response and Lerner grades in patients with AML disease. Graphical illustrations of contingency tables displaying case numbers and overall p-values of fisher´s exact count test. Mean and median grades are categorized in ranges.
(TIF)

S29 Fig Comparison of steroid response and sale grades in patients with AML disease. Graphical illustrations of contingency tables displaying case numbers and overall p-values of fisher´s exact count test. Mean and median grades are categorized in ranges.
(TIF)

S30 Fig Comparison of steroid response and Melson grades in patients with AML disease. Graphical illustrations of contingency tables displaying case numbers and overall p-values of fisher´s exact count test. Mean and median grades are categorized in ranges.
(TIF)

S31 Fig Multivariate generalized linear model of steroid response versus Glucksberg (intestinal GvHD) and Lerner grades in patients with AML disease. Forest plot of regression result depicting case numbers, odds ratios, confidence intervals and p-values.
(TIF)

S32 Fig Univariate COX regression of non-relapse mortality versus steroid application and response in patients with AML disease. Forest plot of regression result depicting case numbers, hazard ratios, confidence intervals and p-values.
(TIF)

S33 Fig Univariate COX regression of overall survival versus steroid application and response in patients with AML disease. Forest plot of regression result depicting case numbers, hazard ratios, confidence intervals and p-values.
(TIF)

S34 Fig Univariate COX regression of overall survival and non-relapse mortality versus biopsy count in patients with AML disease. Forest plot of regression result depicting case numbers, hazard ratios, confidence intervals and p-values.
(TIF)
