# Supplementary figures and images for: An investigation of the diagnostic, predictive, and prognostic impacts of three colonic biopsy grading systems for acute graft versus host disease
Source: PLoS One. 2021 Aug 26;16(8):e0256543. doi: 10.1371/journal.pone.0256543 (PMC8389423; doi:10.1371/journal.pone.0256543)

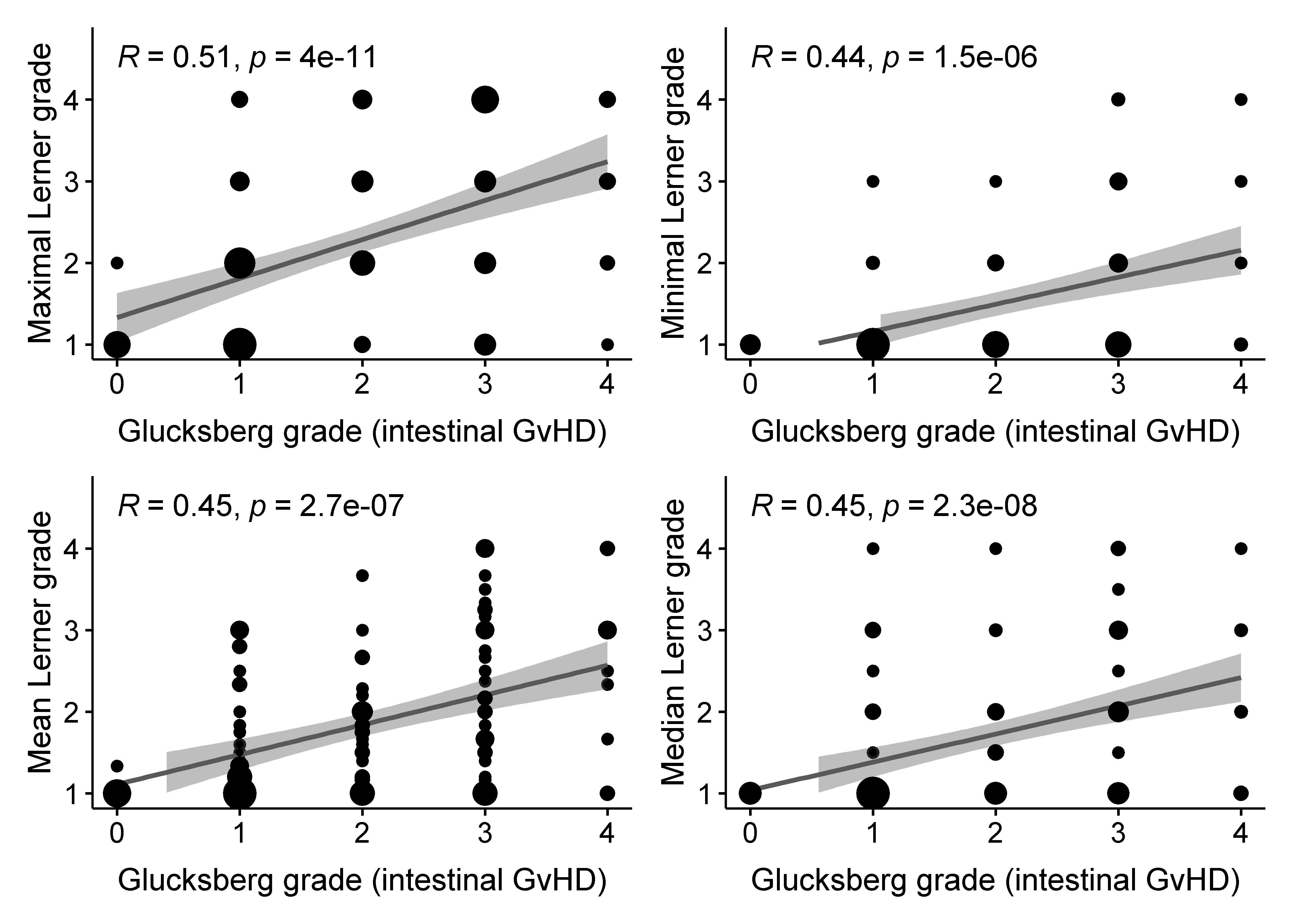

Supplement: S1 Fig — Diameters of dots correspond to case numbers linearly. Light grey bands symbolize 95% confidence intervals and dark grey lines linear regression lines. (TIF) [file pone.0256543.s002.tif]

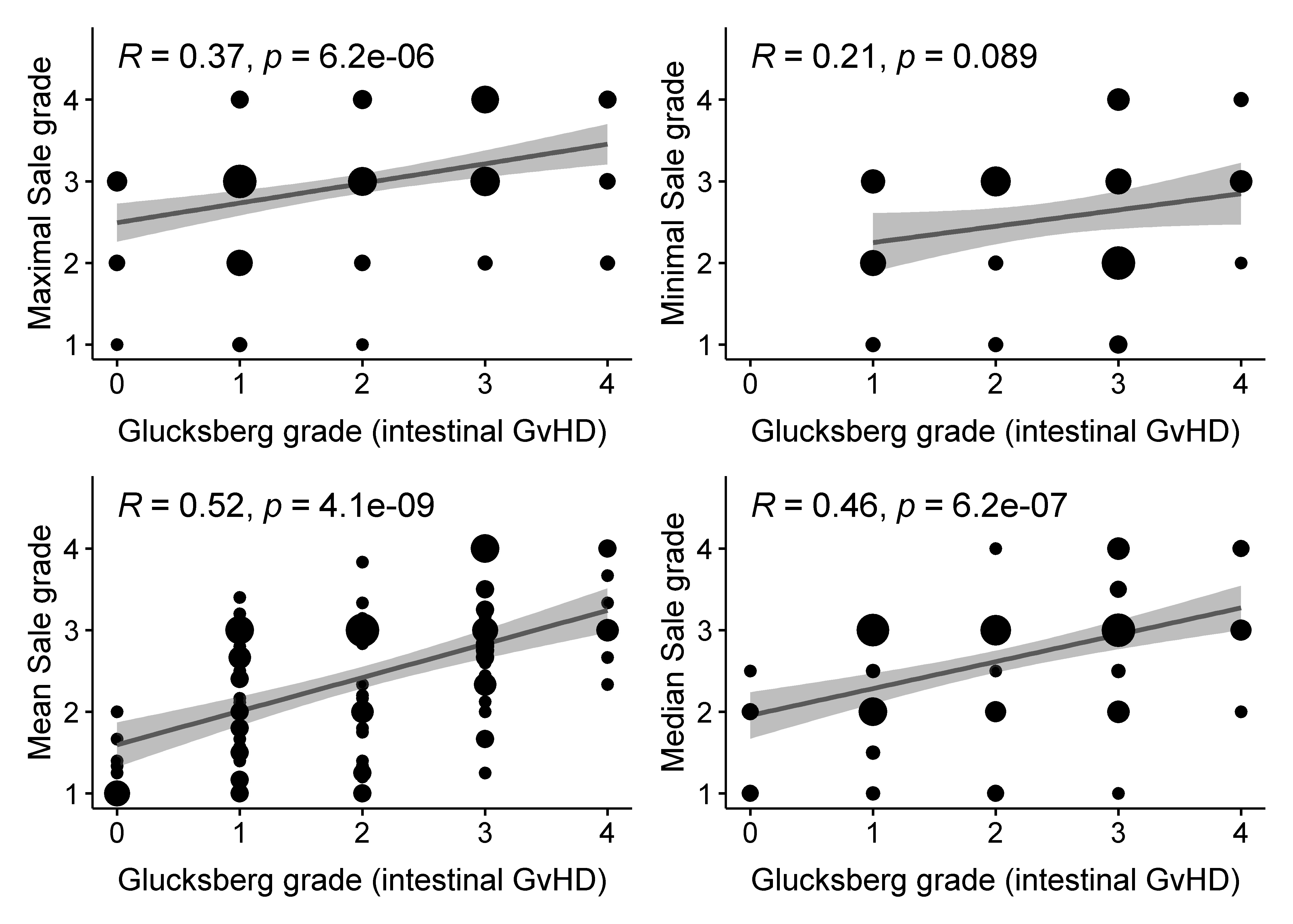

Supplement: S2 Fig — Diameters of dots correspond to case numbers linearly. Light grey bands symbolize 95% confidence intervals and dark grey lines linear regression lines. (TIF) [file pone.0256543.s003.tif]

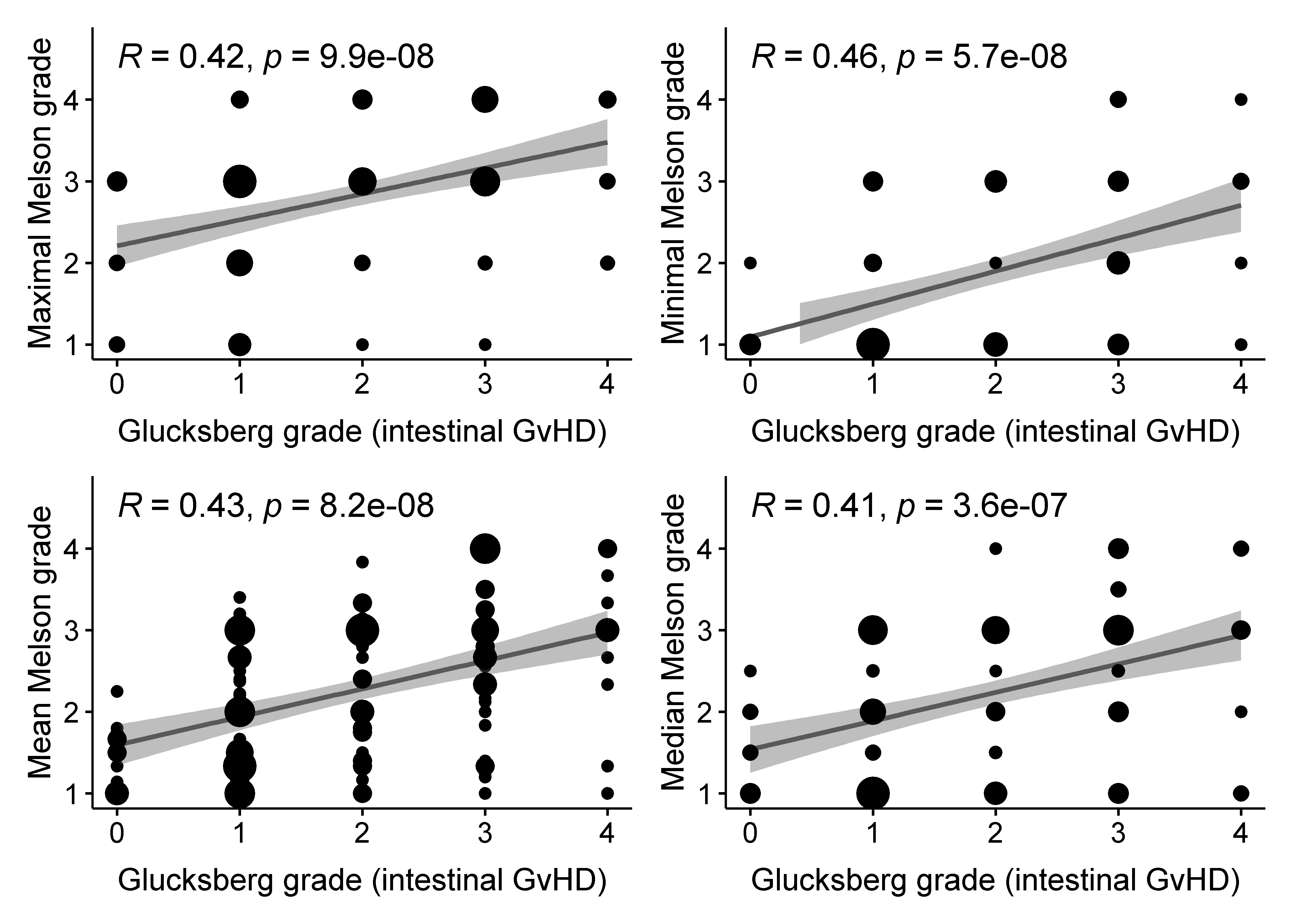

Supplement: S3 Fig — Diameters of dots correspond to case numbers linearly. Light grey bands symbolize 95% confidence intervals and dark grey lines linear regression lines. (TIF) [file pone.0256543.s004.tif]

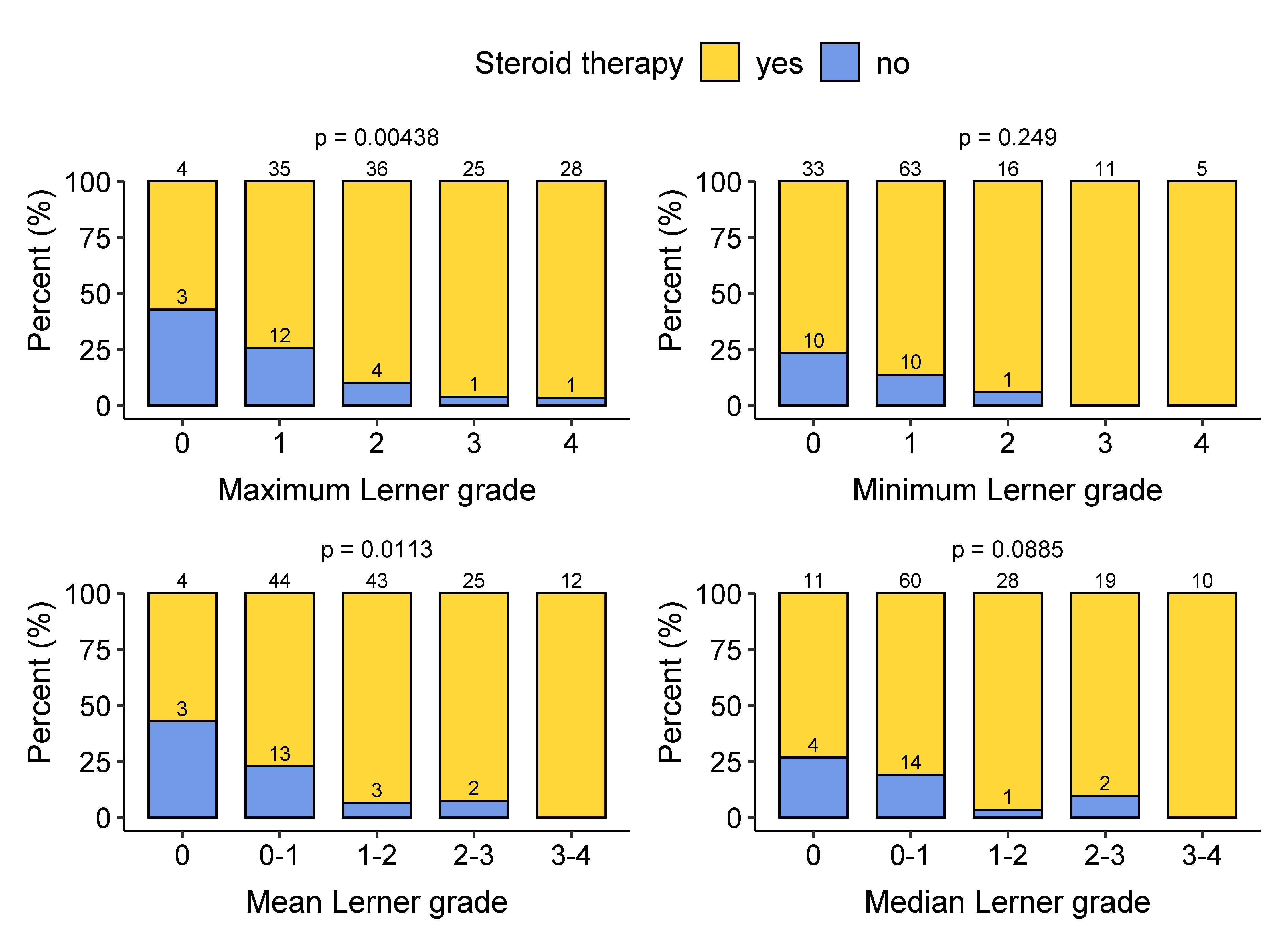

Supplement: S4 Fig — Graphical illustrations of contingency tables displaying case numbers and overall p-values of fisher´s exact count test. Mean and median grades are categorized in ranges. (TIF) [file pone.0256543.s005.tif]

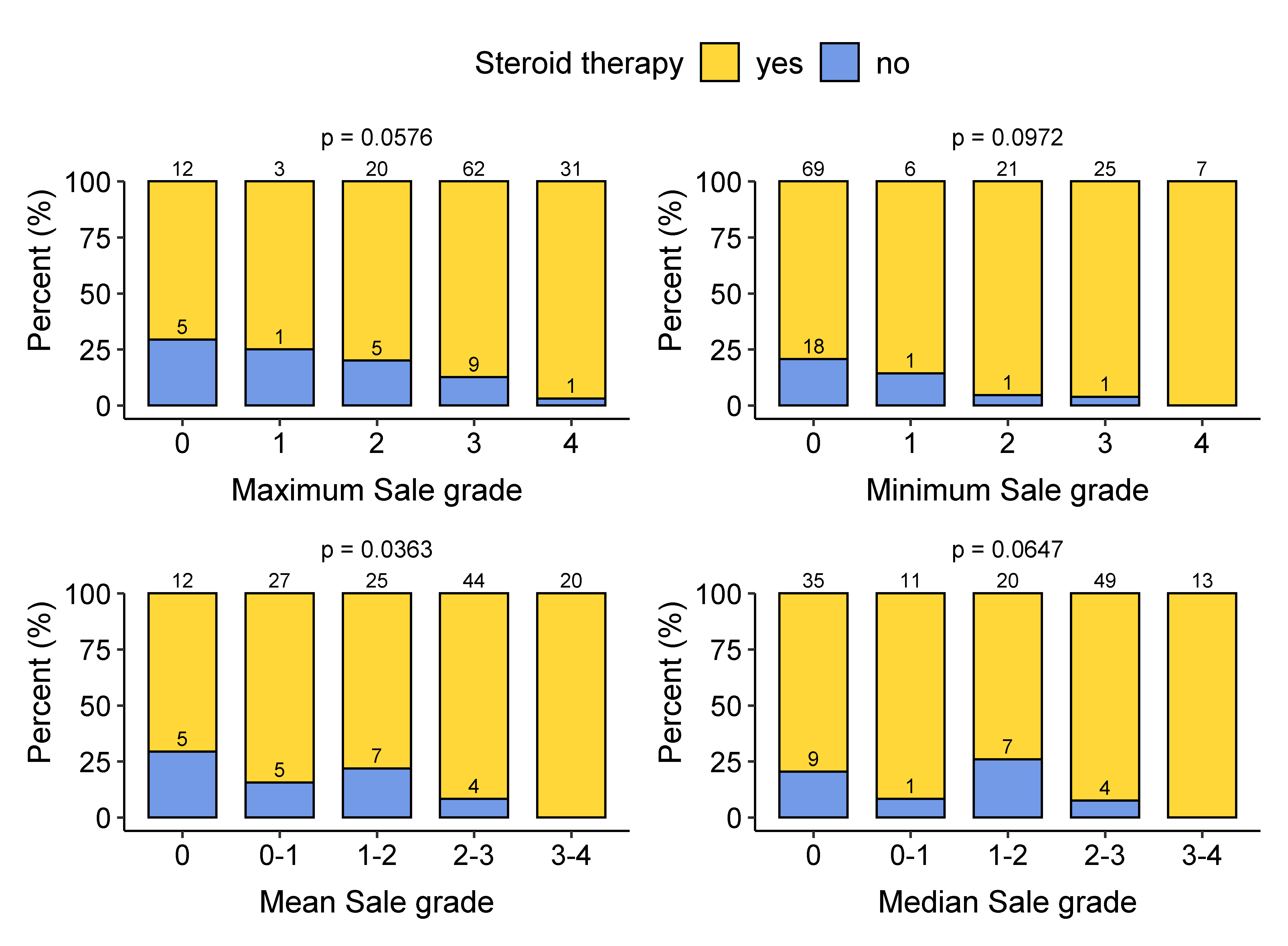

Supplement: S5 Fig — Graphical illustrations of contingency tables displaying case numbers and overall p-values of fisher´s exact count test. Mean and median grades are categorized in ranges. (TIF) [file pone.0256543.s006.tif]

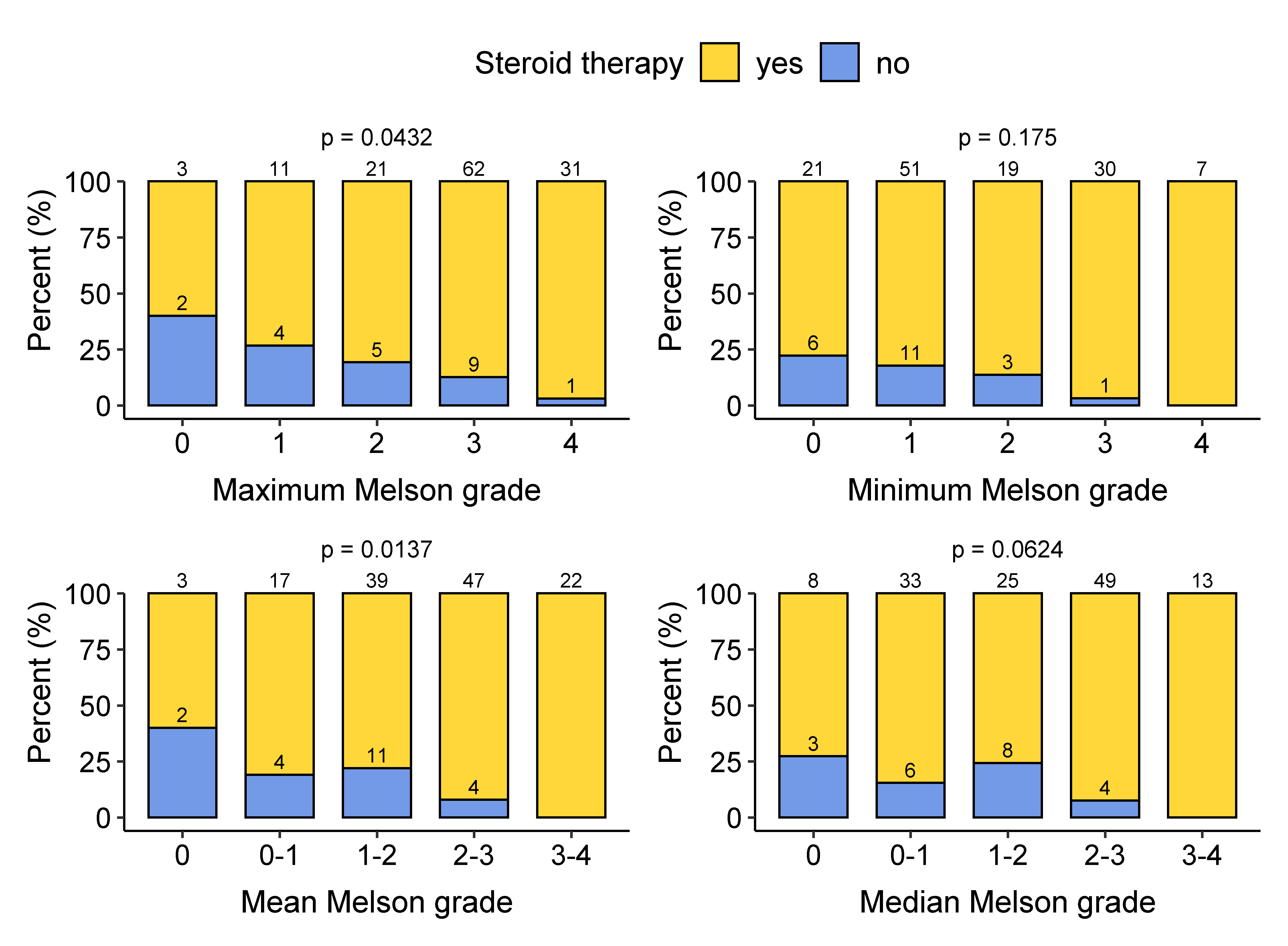

Supplement: S6 Fig — Graphical illustrations of contingency tables displaying case numbers and overall p-values of fisher´s exact count test. Mean and median grades are categorized in ranges. (TIF) [file pone.0256543.s007.tif]

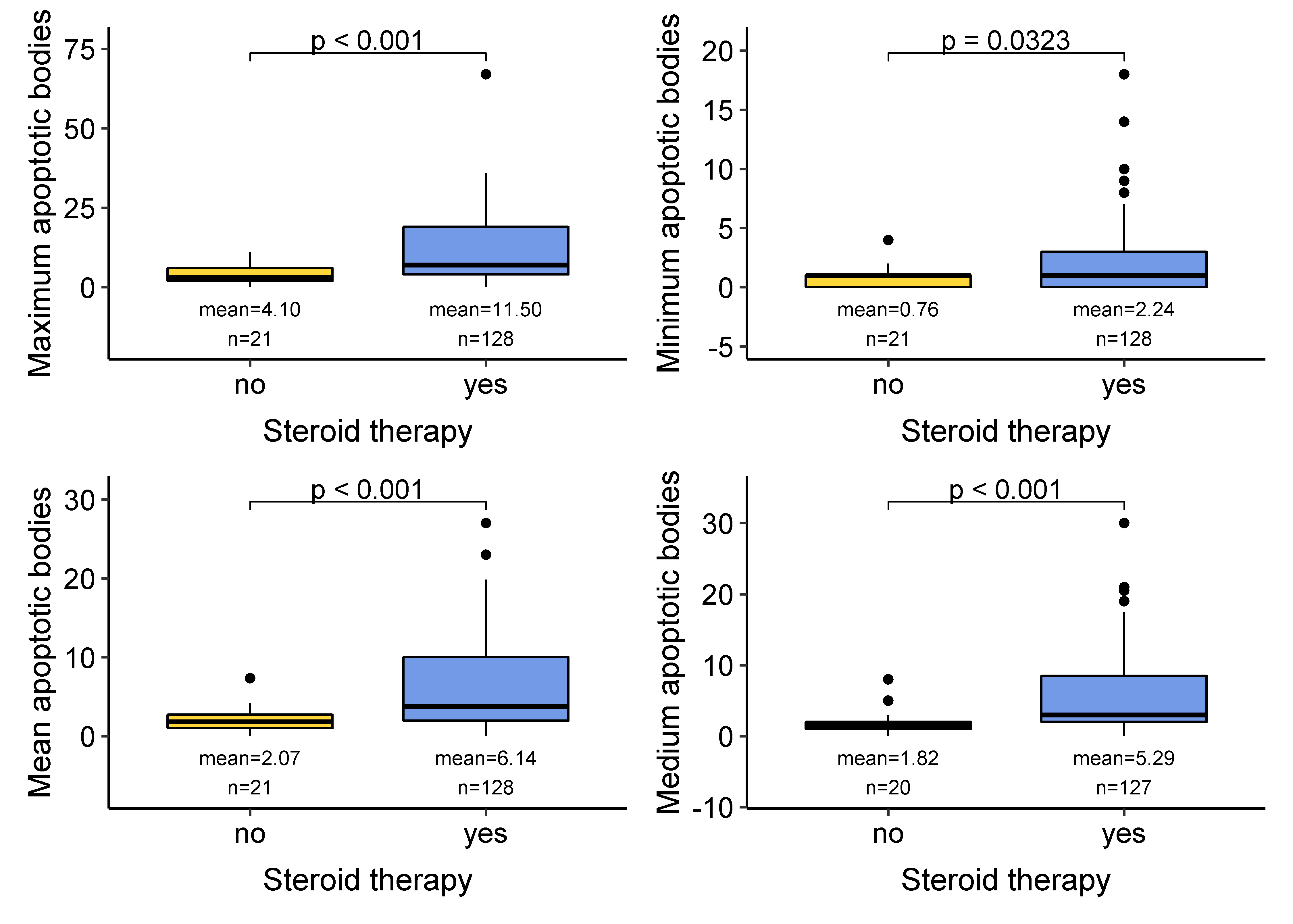

Supplement: S7 Fig — Box plots showing differences between amount of apoptotic bodies and steroid therapy. Displayed p-values derived from unpaired Mann-Whitney U-test. (TIF) [file pone.0256543.s008.tif]

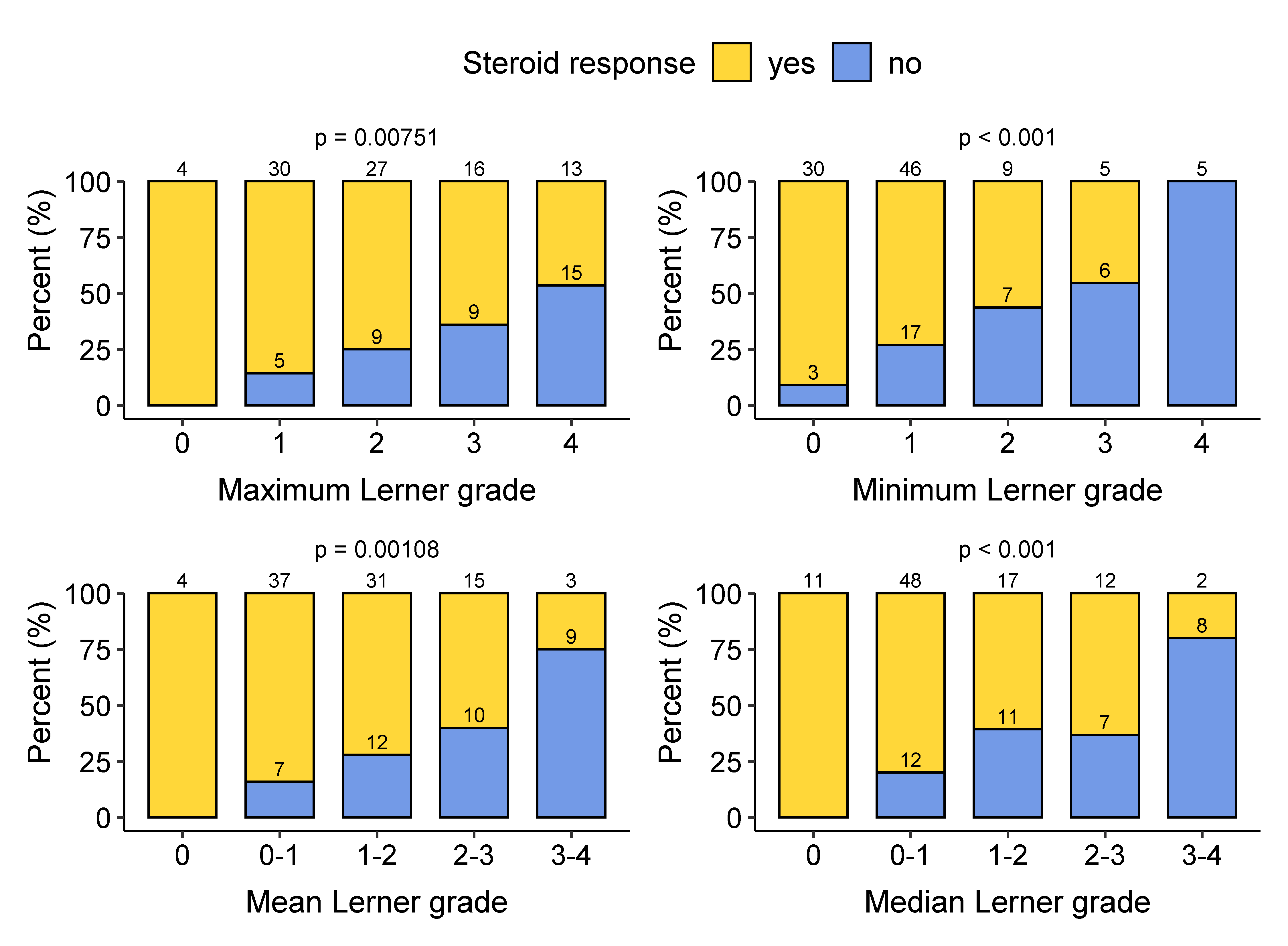

Supplement: S8 Fig — Graphical illustrations of contingency tables displaying case numbers and overall p-values of fisher´s exact count test. Mean and median grades are categorized in ranges. (TIF) [file pone.0256543.s009.tif]

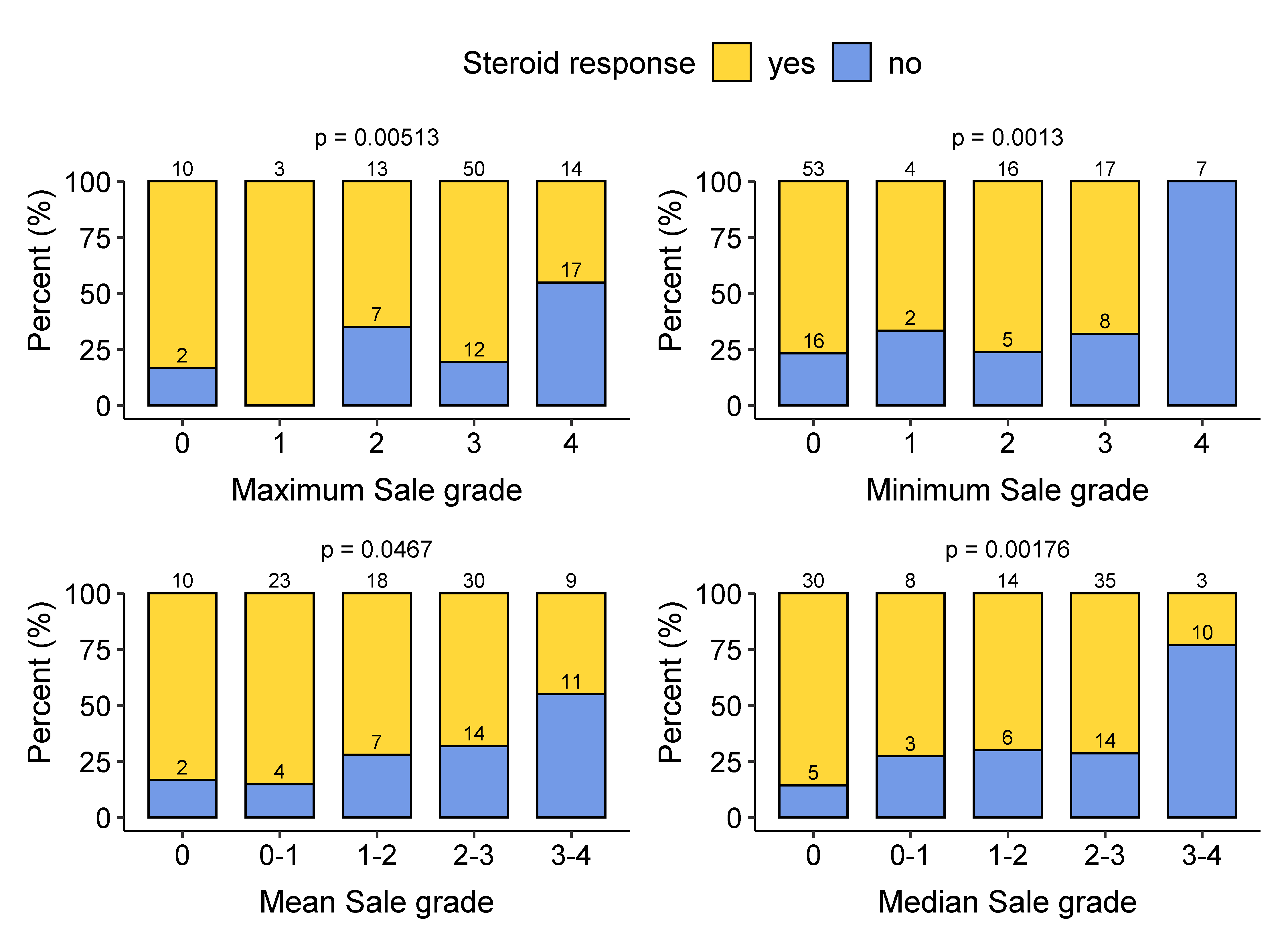

Supplement: S9 Fig — Graphical illustrations of contingency tables displaying case numbers and overall p-values of fisher´s exact count test. Mean and median grades are categorized in ranges. (TIF) [file pone.0256543.s010.tif]

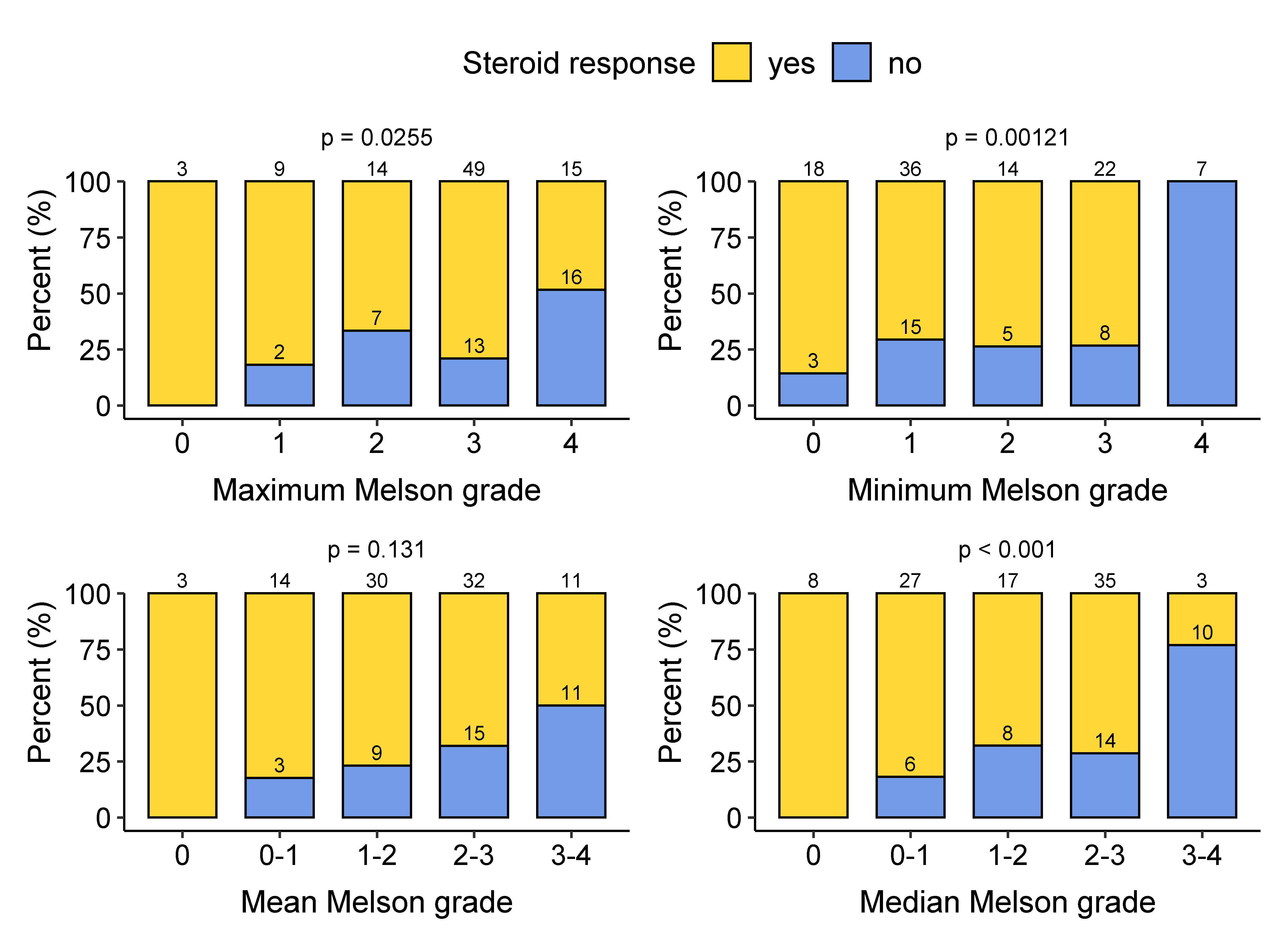

Supplement: S10 Fig — Graphical illustrations of contingency tables displaying case numbers and overall p-values of fisher´s exact count test. Mean and median grades are categorized in ranges. (TIF) [file pone.0256543.s011.tif]

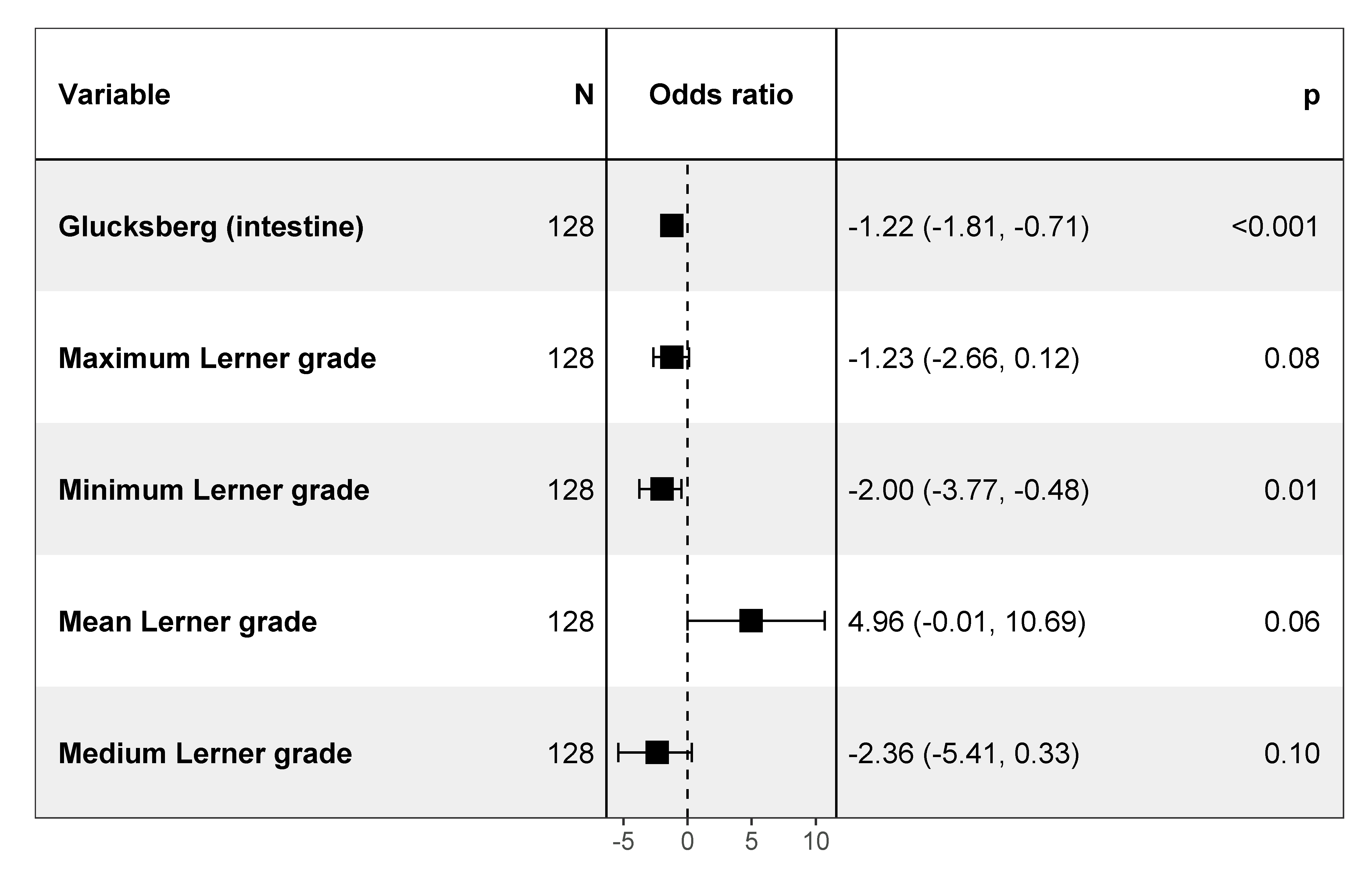

Supplement: S11 Fig — Forest plot of regression result depicting case numbers, odds ratios, confidence intervals and p-values. (TIF) [file pone.0256543.s012.tif]

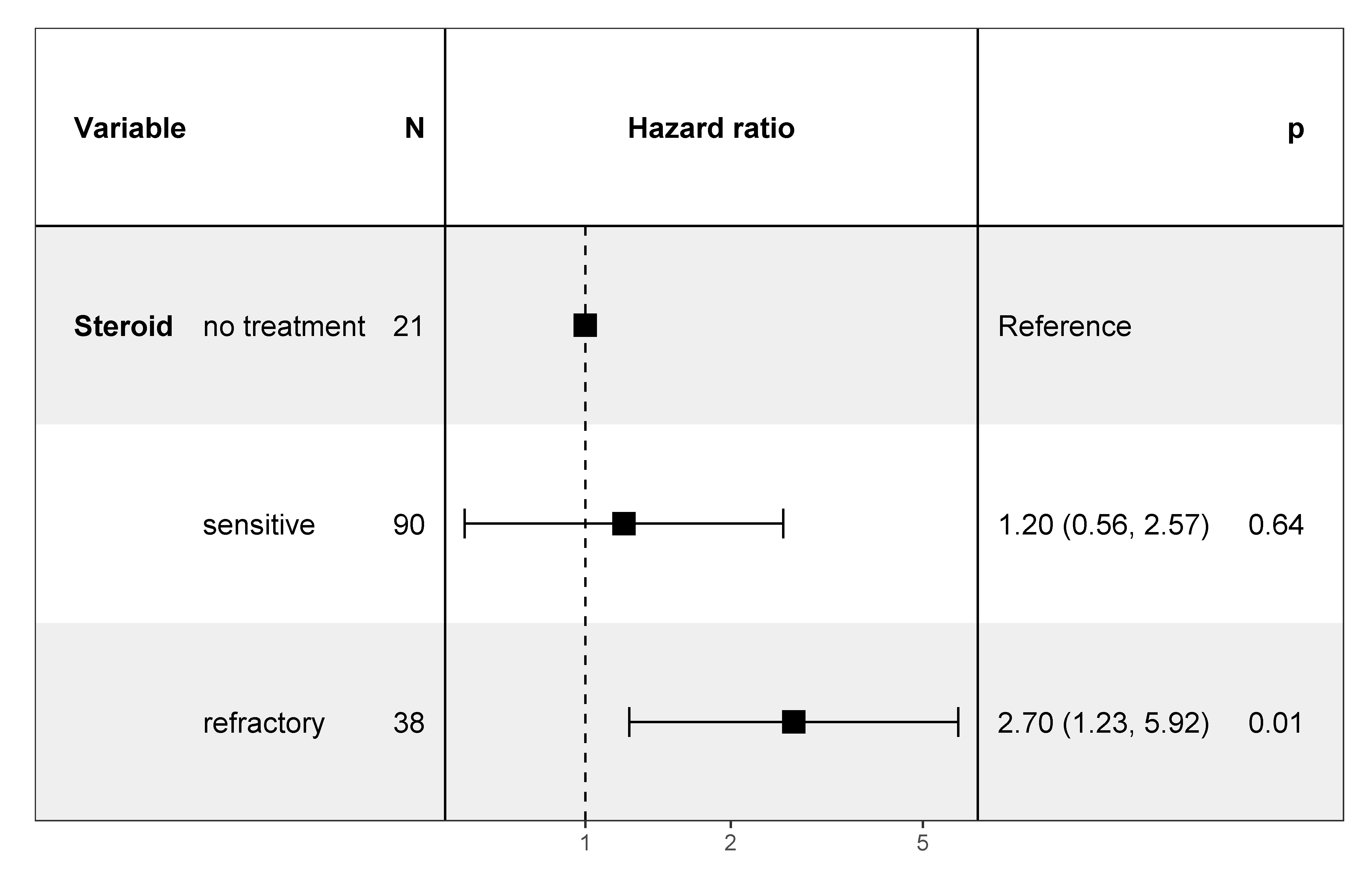

Supplement: S12 Fig — Forest plot of regression result depicting case numbers, hazard ratios, confidence intervals and p-values. (TIF) [file pone.0256543.s013.tif]

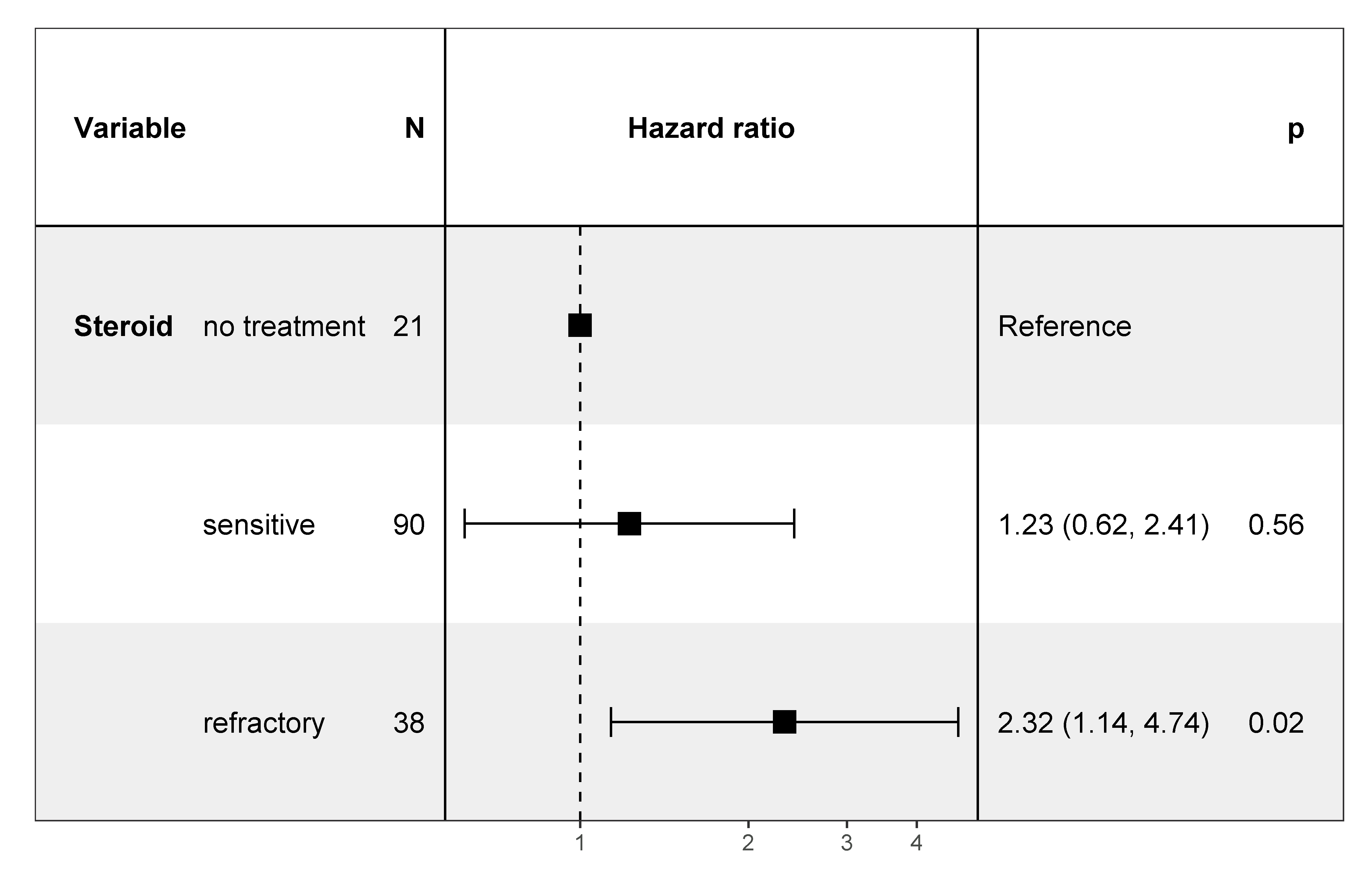

Supplement: S13 Fig — Forest plot of regression result depicting case numbers, hazard ratios, confidence intervals and p-values. (TIF) [file pone.0256543.s014.tif]

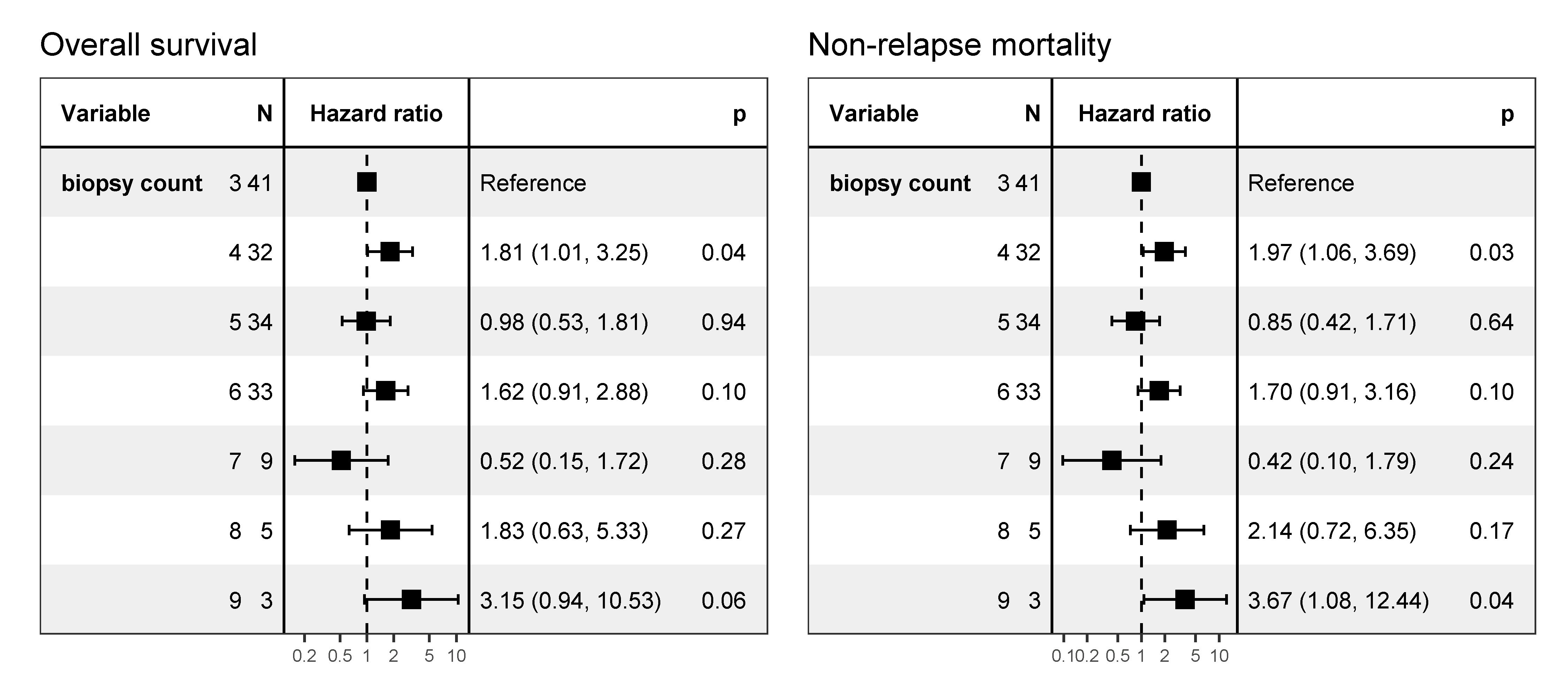

Supplement: S14 Fig — Forest plot of regression result depicting case numbers, hazard ratios, confidence intervals and p-values. (TIF) [file pone.0256543.s015.tif]

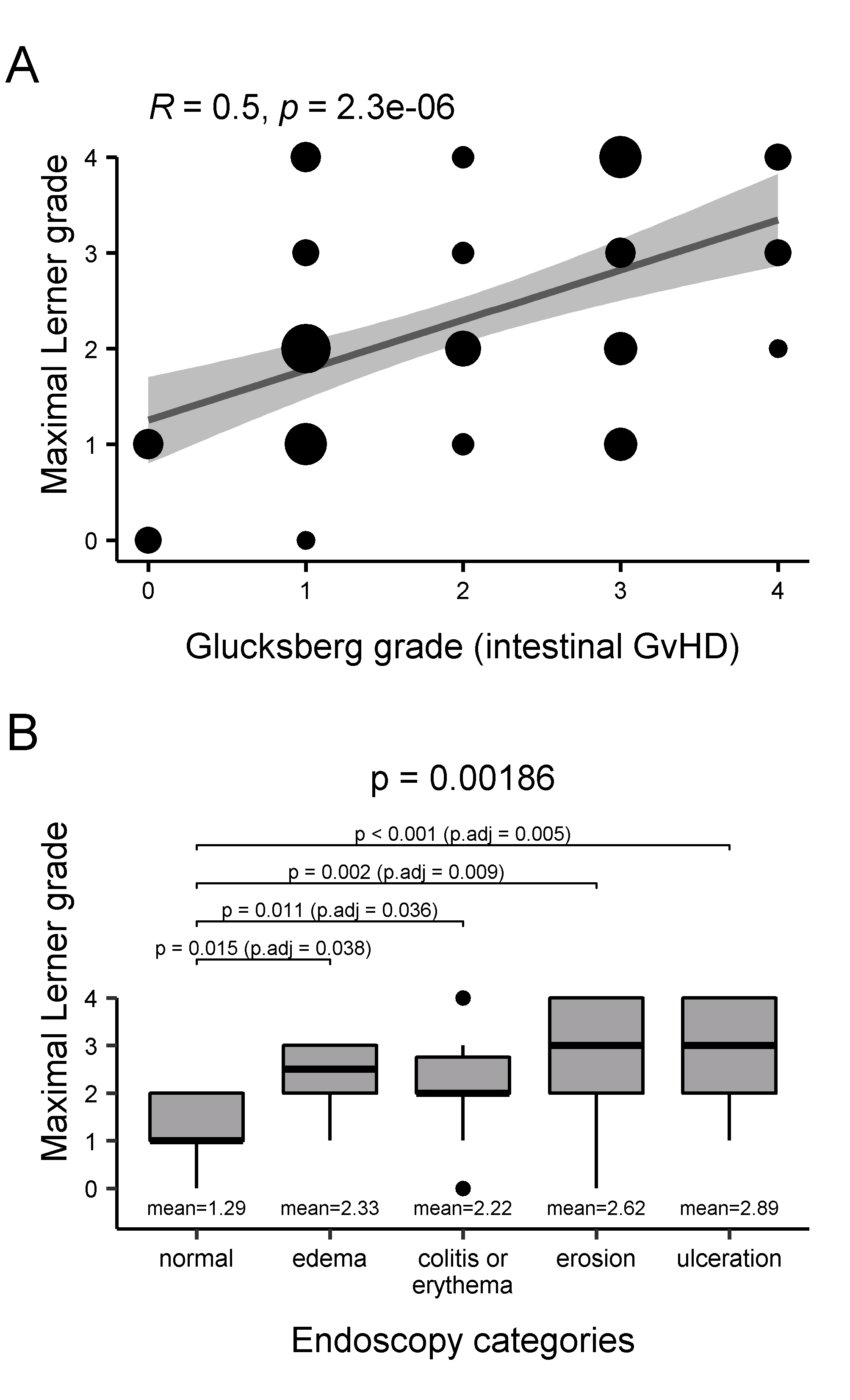

Supplement: S15 Fig — Diameters of dots correlate with number of cases linearly (A). Endoscopic findings in patients with acute GvHD (B). (TIF) [file pone.0256543.s016.tif]

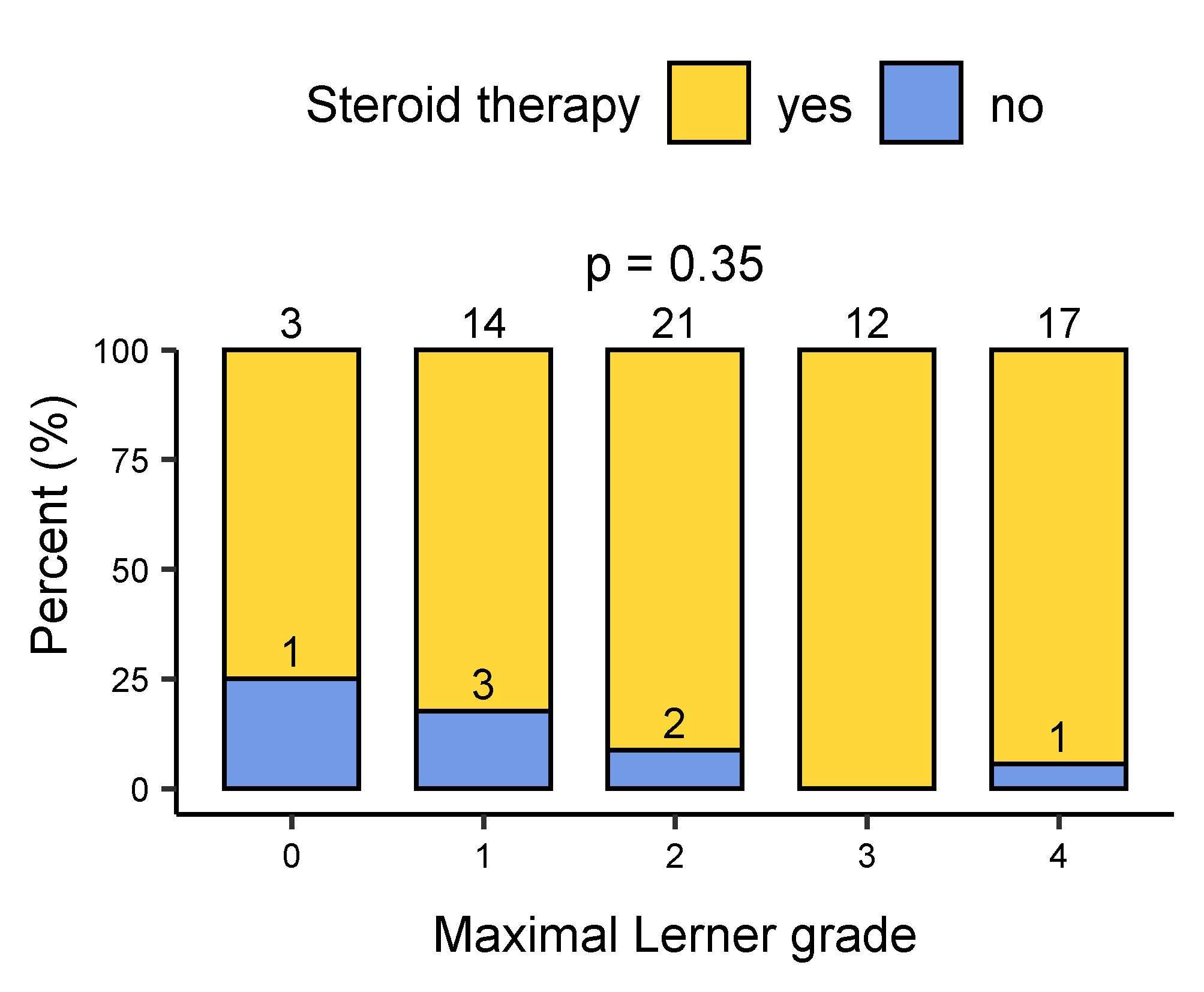

Supplement: S16 Fig — Graphical illustration of contingency table displaying case numbers and overall p-value of fisher´s exact count test. (TIF) [file pone.0256543.s017.tif]

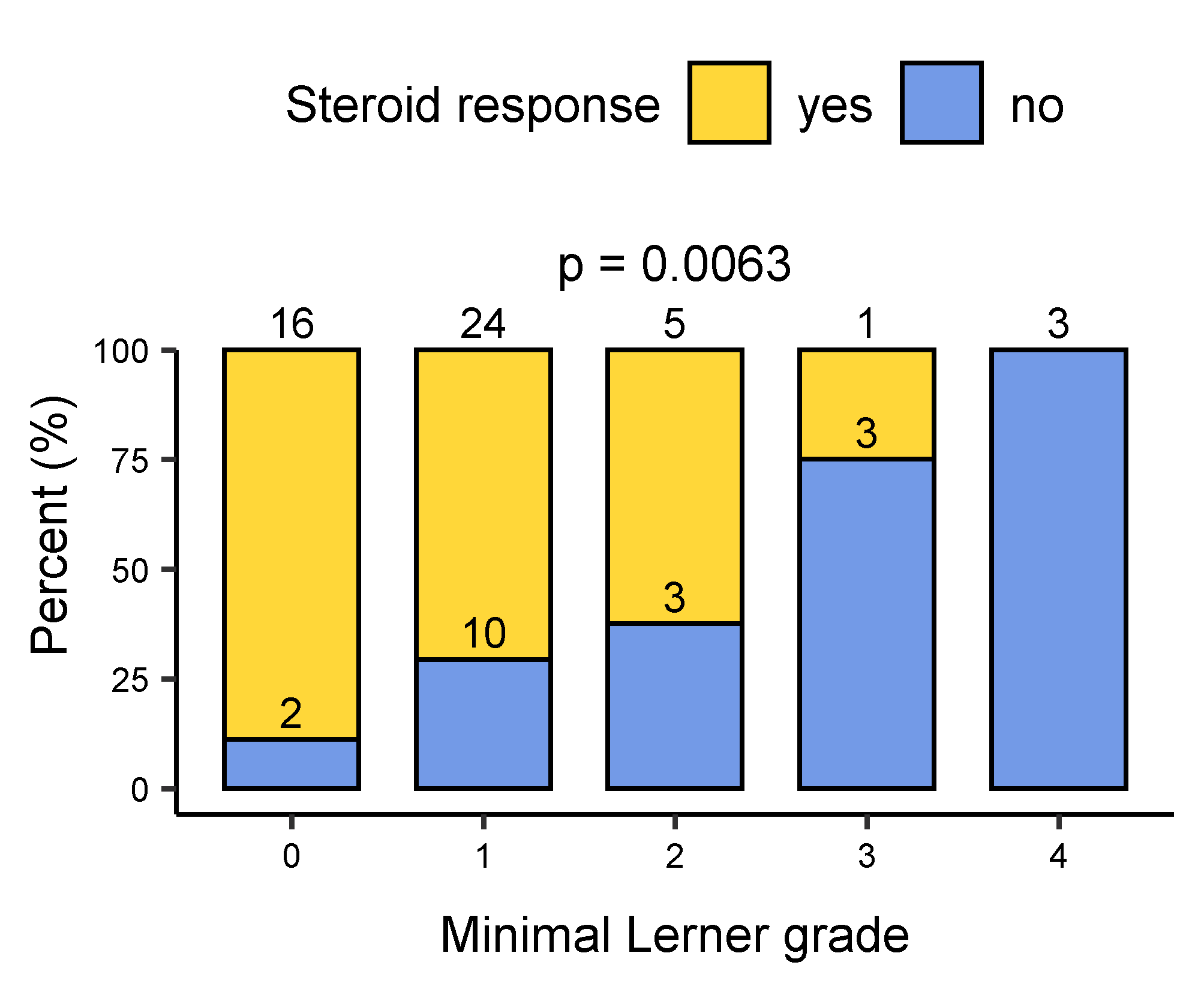

Supplement: S17 Fig — Graphical illustration of contingency table displaying case numbers and overall p-value of fisher´s exact count test. (TIF) [file pone.0256543.s018.tif]

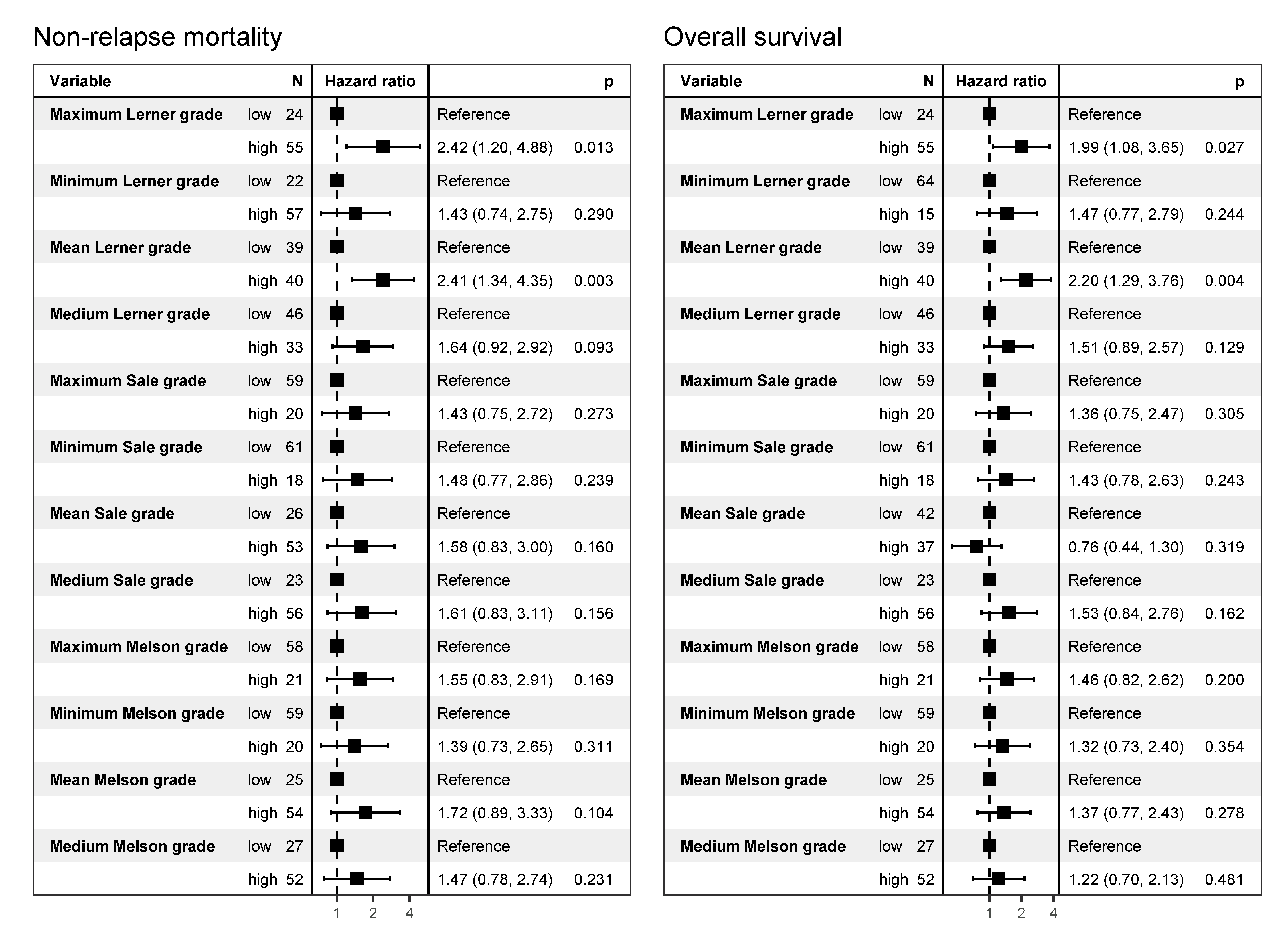

Supplement: S18 Fig — Forest plots of regression results depicting case numbers, hazard ratios, confidence intervals and p-values. (TIF) [file pone.0256543.s019.tif]

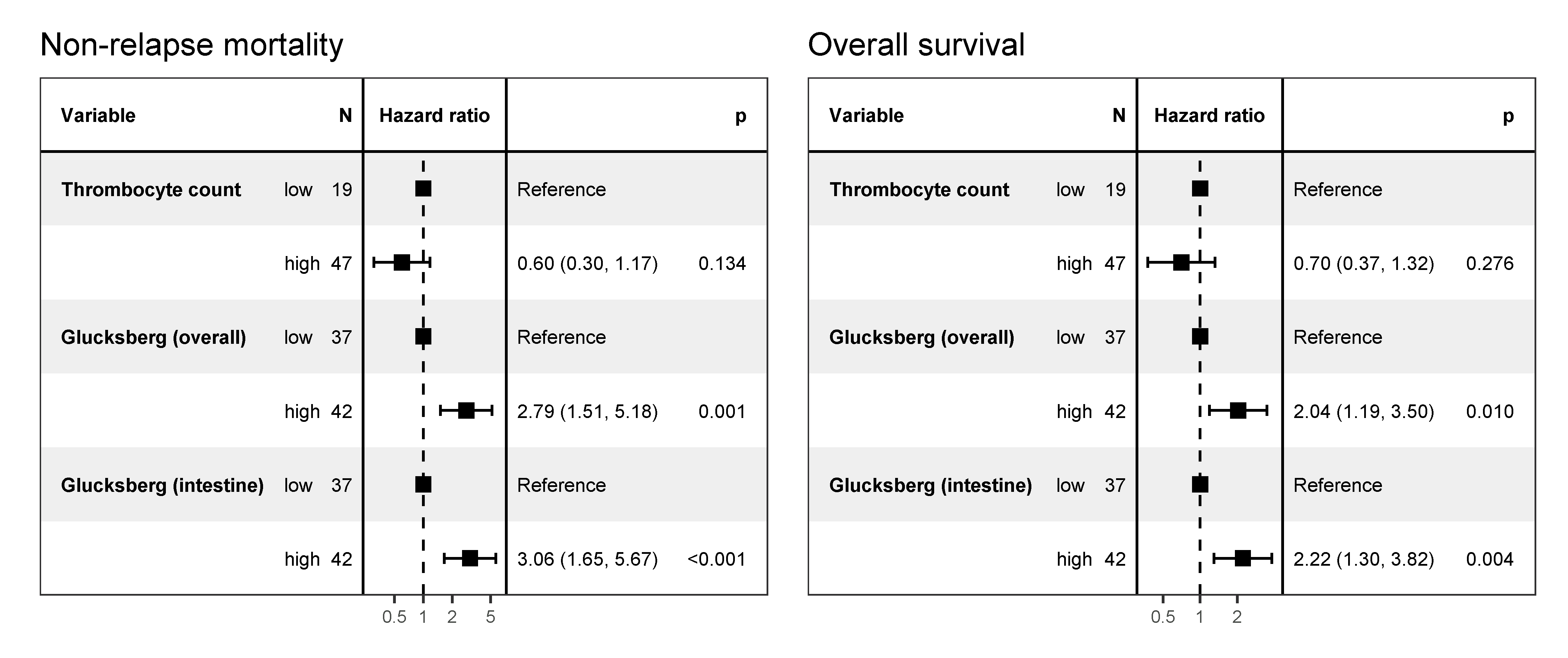

Supplement: S19 Fig — Forest plots of regression results depicting case numbers, hazard ratios, confidence intervals and p-values. (TIF) [file pone.0256543.s020.tif]

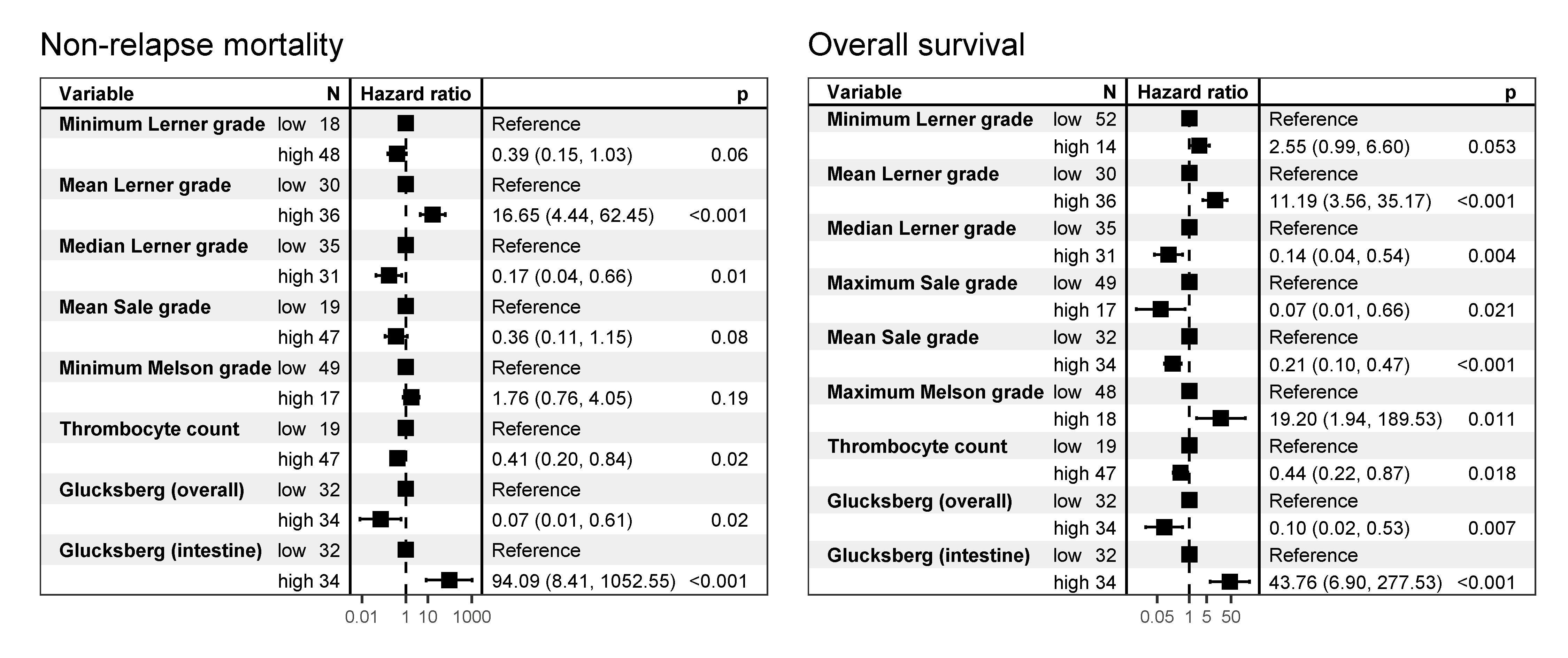

Supplement: S20 Fig — Forest plots of regression results depicting case numbers, hazard ratios, confidence intervals and p-values. (TIF) [file pone.0256543.s021.tif]

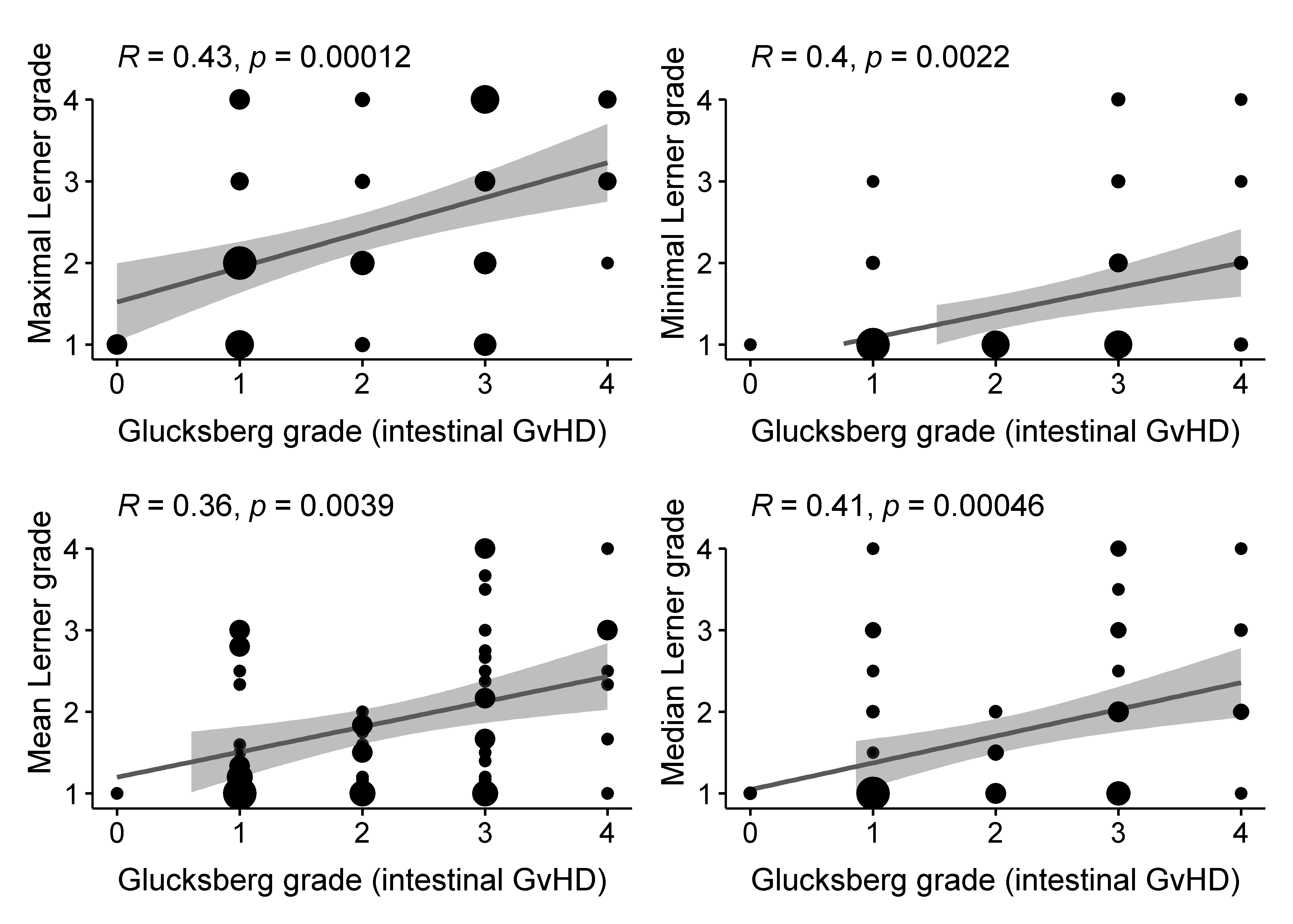

Supplement: S21 Fig — Diameters of dots correspond to case numbers linearly. Light grey bands symbolize 95% confidence intervals and dark grey lines linear regression lines. (TIF) [file pone.0256543.s022.tif]

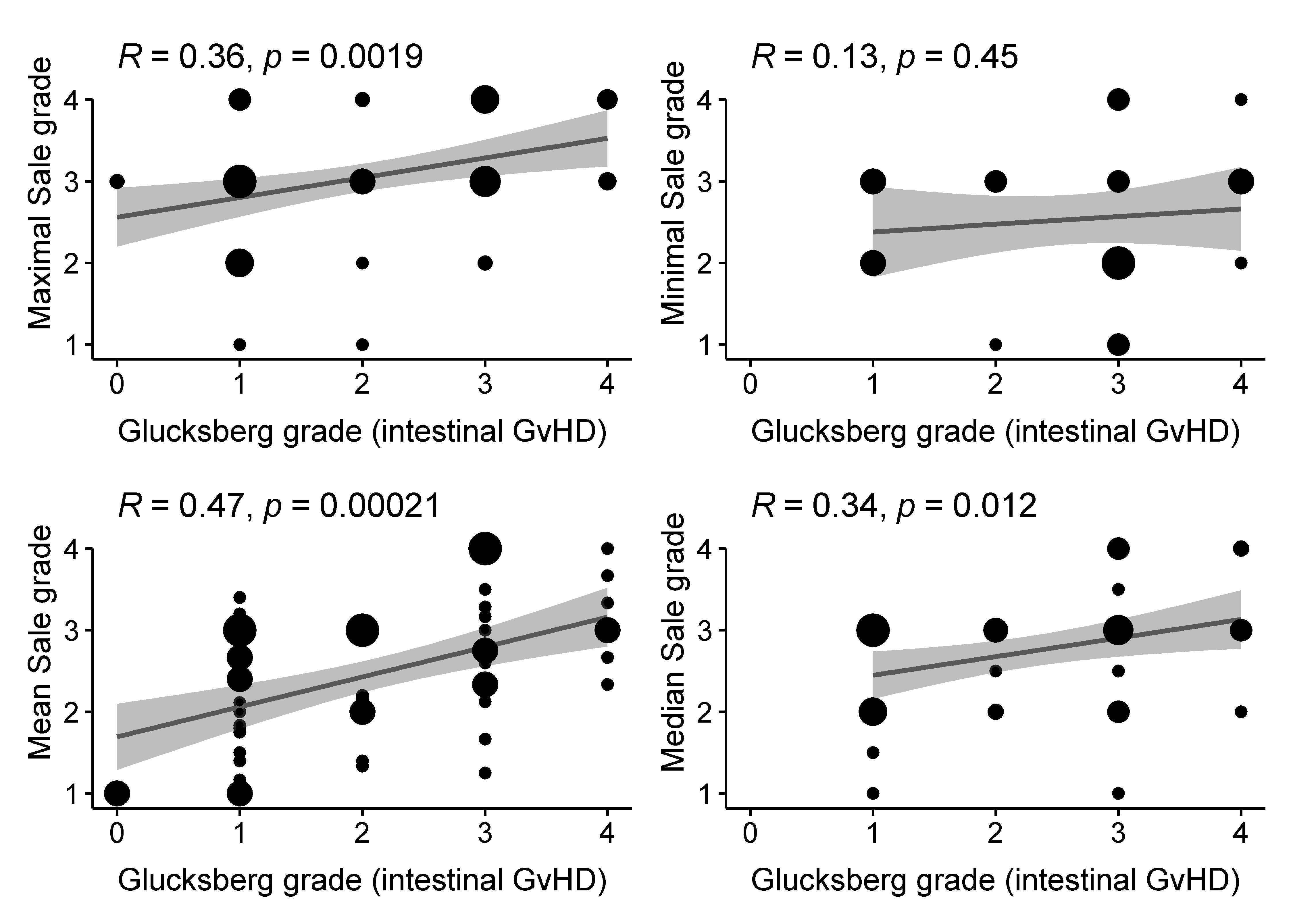

Supplement: S22 Fig — Diameters of dots correspond to case numbers linearly. Light grey bands symbolize 95% confidence intervals and dark grey lines linear regression lines. (TIF) [file pone.0256543.s023.tif]

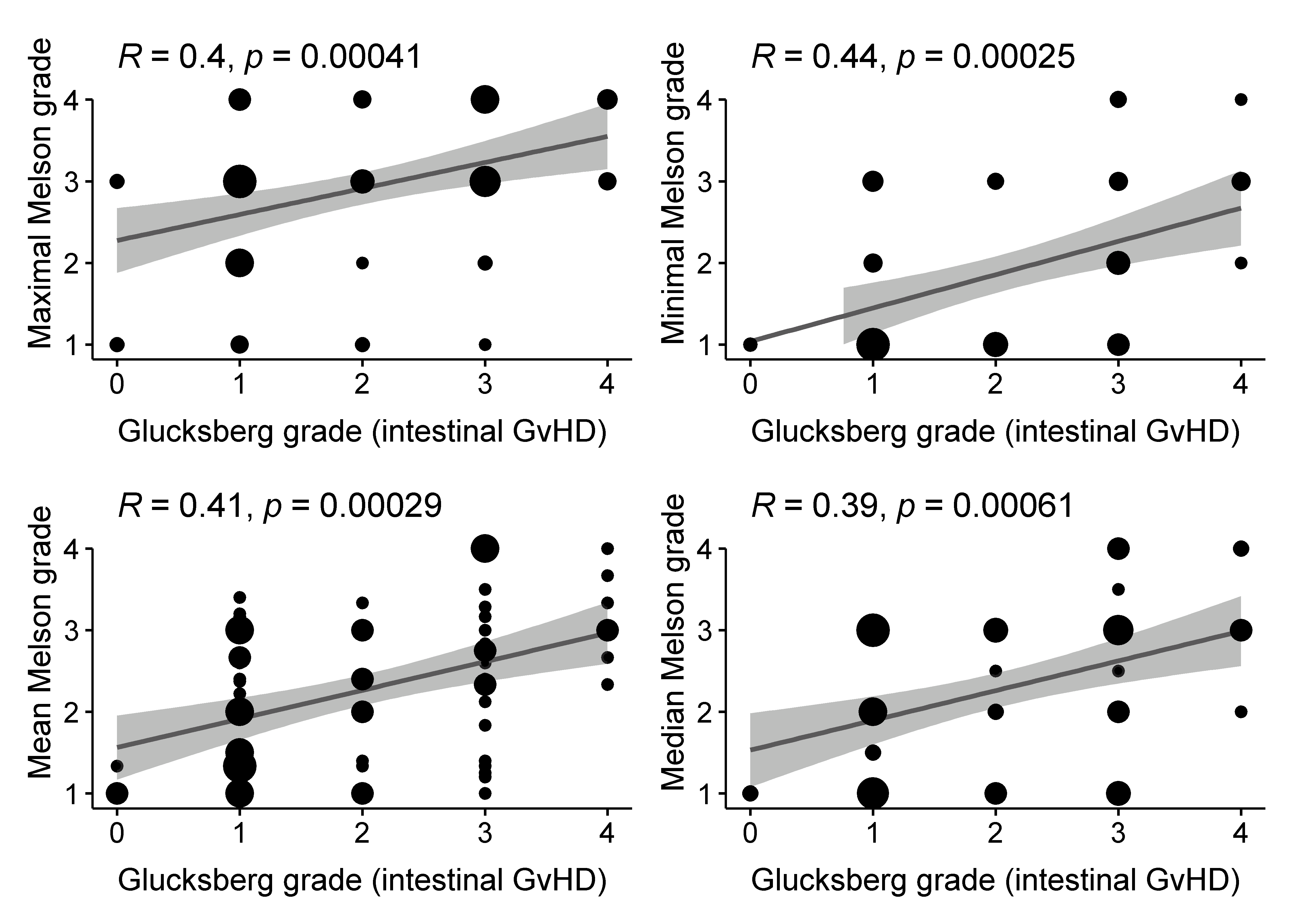

Supplement: S23 Fig — Diameters of dots correspond to case numbers linearly. Light grey bands symbolize 95% confidence intervals and dark grey lines linear regression lines. (TIF) [file pone.0256543.s024.tif]

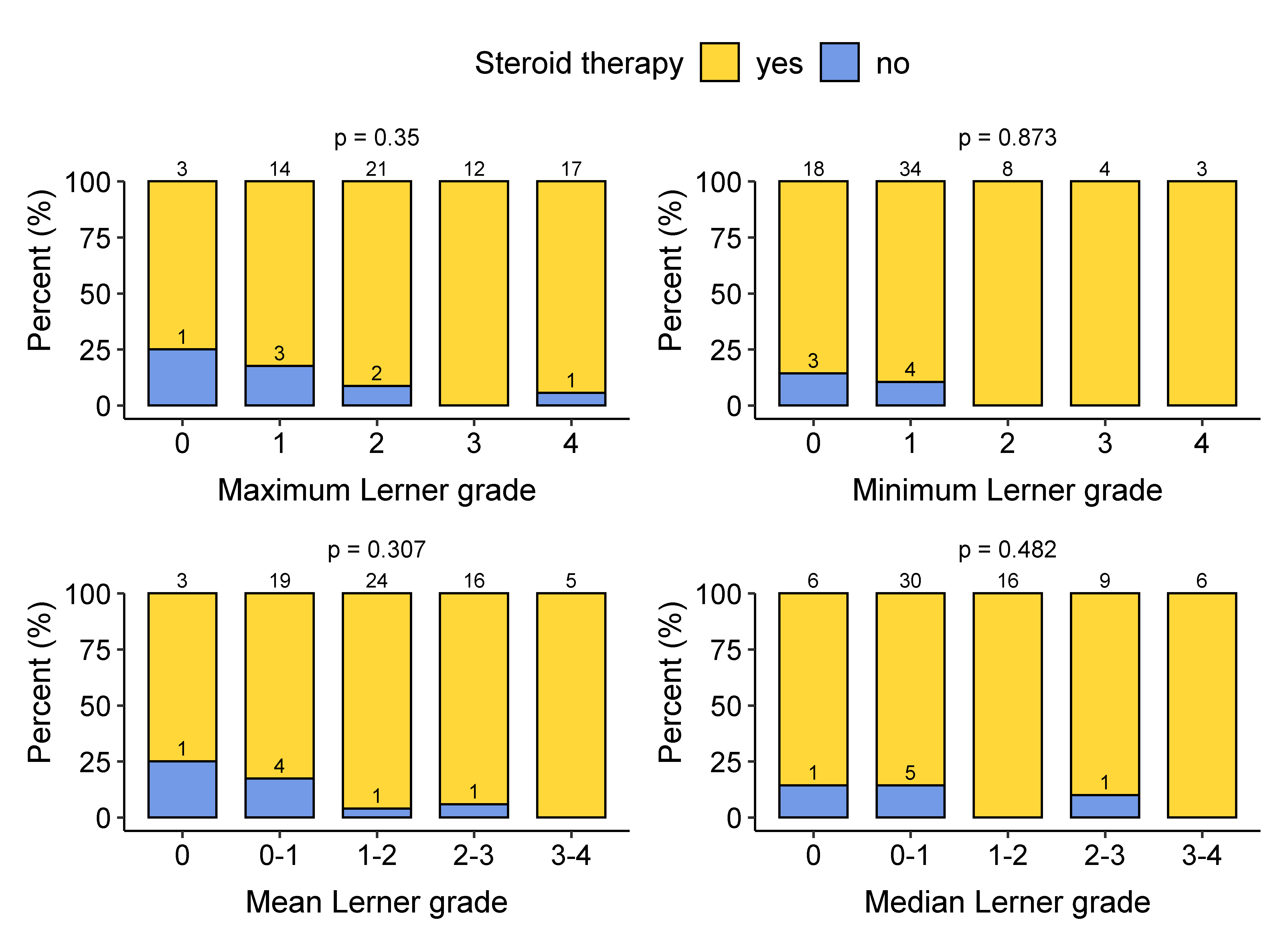

Supplement: S24 Fig — Graphical illustrations of contingency tables displaying case numbers and overall p-values of fisher´s exact count test. Mean and median grades are categorized in ranges. (TIF) [file pone.0256543.s025.tif]

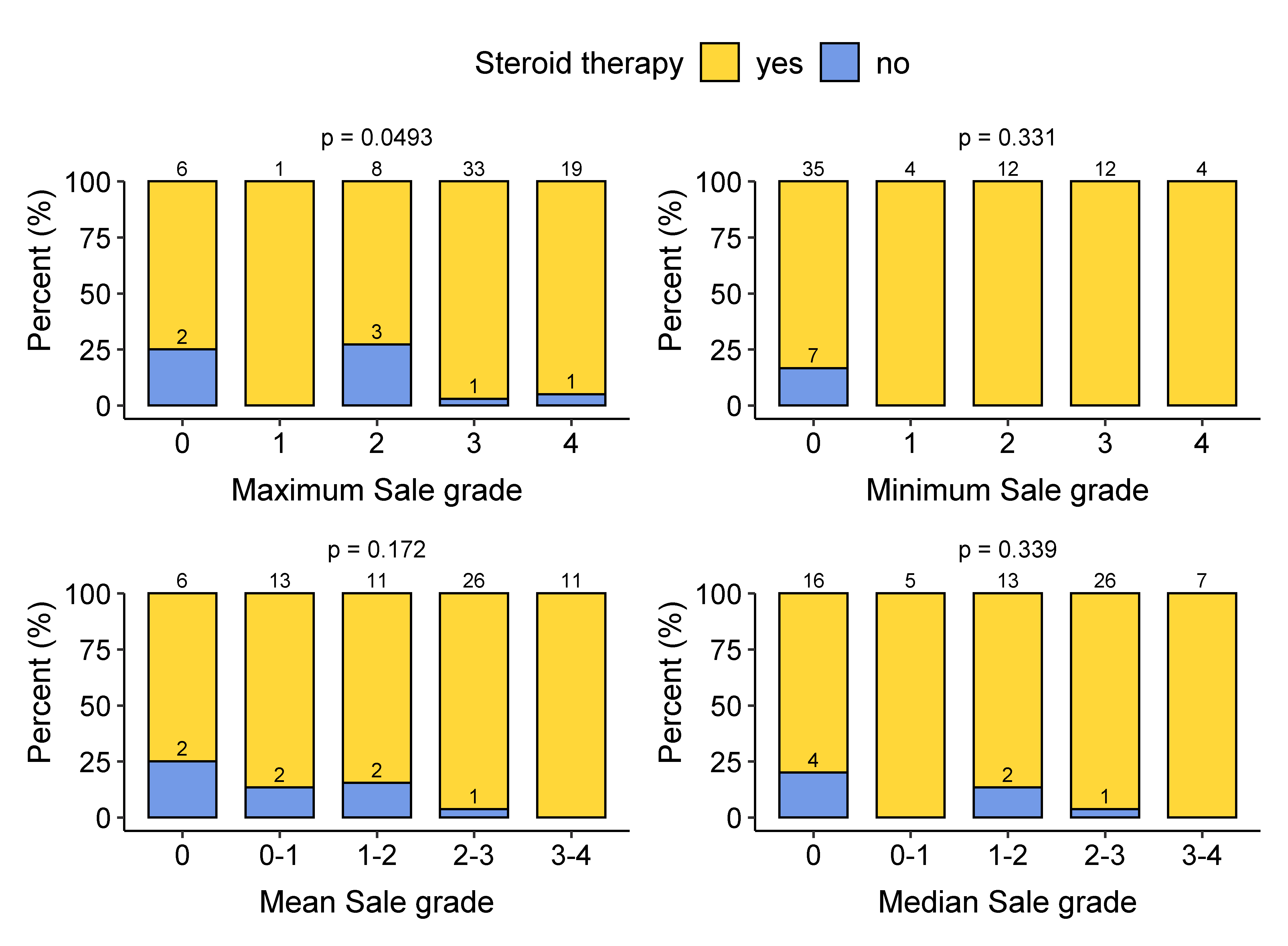

Supplement: S25 Fig — Graphical illustrations of contingency tables displaying case numbers and overall p-values of fisher´s exact count test. Mean and median grades are categorized in ranges. (TIF) [file pone.0256543.s026.tif]

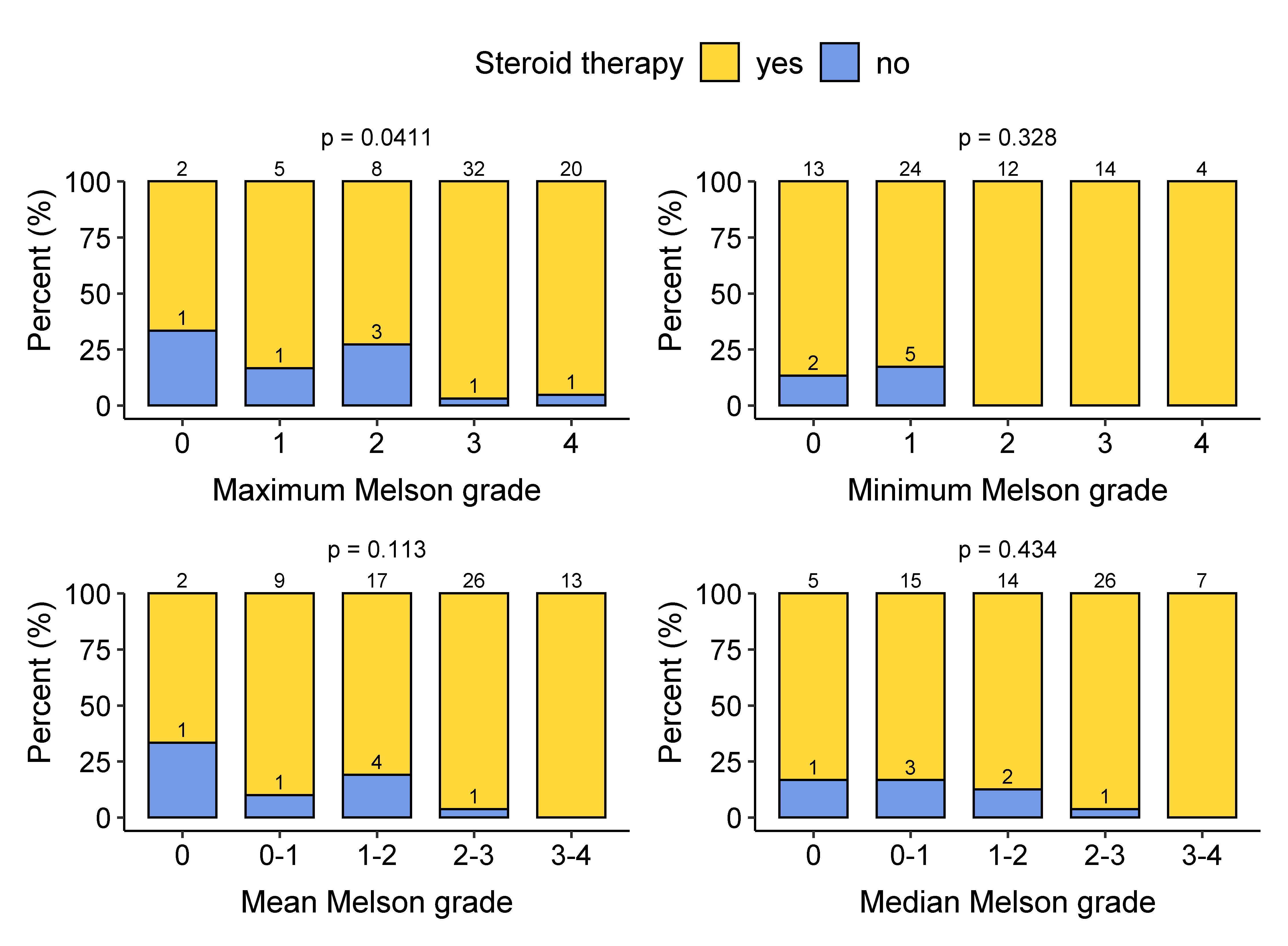

Supplement: S26 Fig — Graphical illustrations of contingency tables displaying case numbers and overall p-values of fisher´s exact count test. Mean and median grades are categorized in ranges. (TIF) [file pone.0256543.s027.tif]

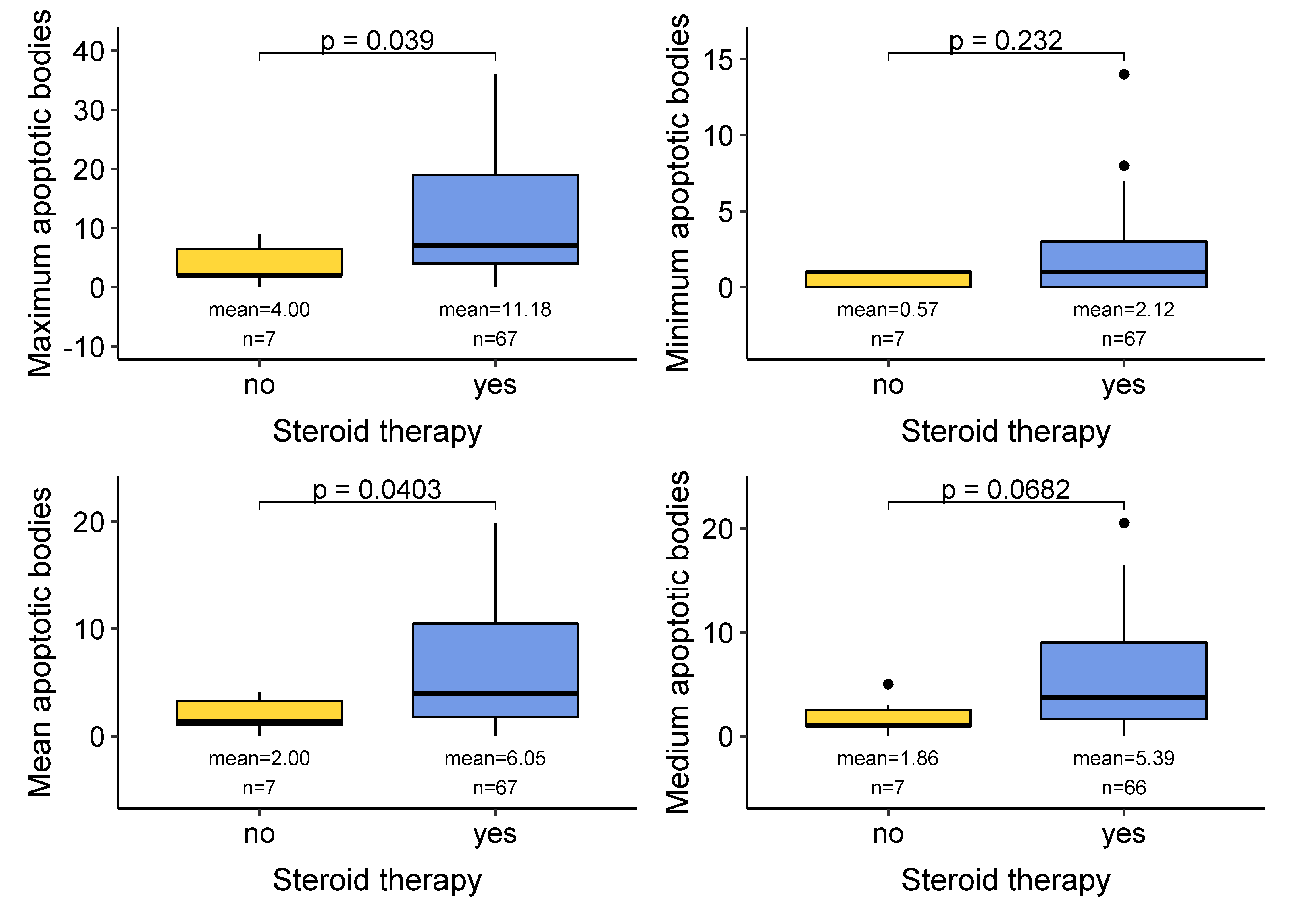

Supplement: S27 Fig — Box plots showing differences between amount of apoptotic bodies and steroid therapy. Displayed p-values derived from unpaired Mann-Whitney U-test. (TIF) [file pone.0256543.s028.tif]

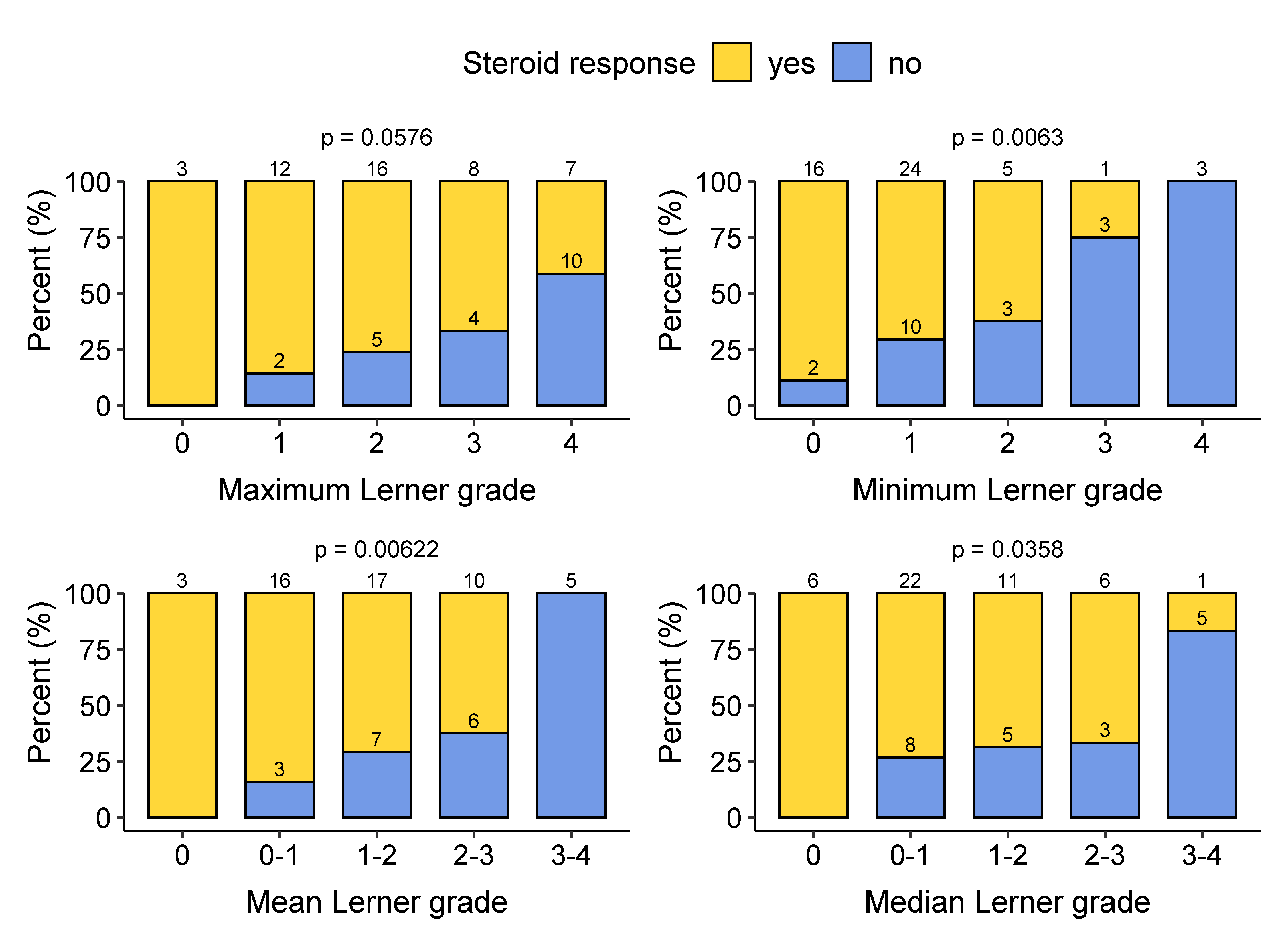

Supplement: S28 Fig — Graphical illustrations of contingency tables displaying case numbers and overall p-values of fisher´s exact count test. Mean and median grades are categorized in ranges. (TIF) [file pone.0256543.s029.tif]

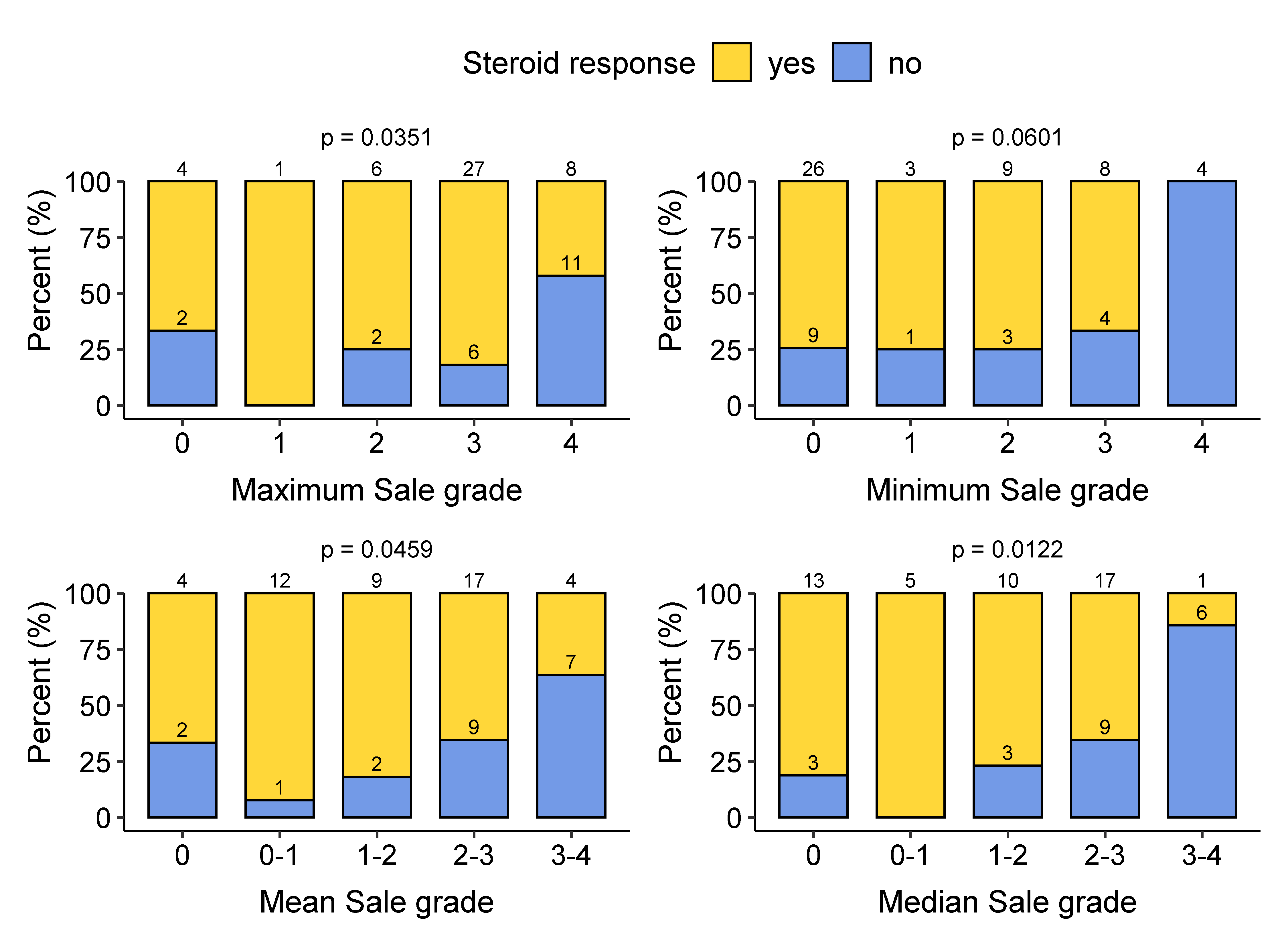

Supplement: S29 Fig — Graphical illustrations of contingency tables displaying case numbers and overall p-values of fisher´s exact count test. Mean and median grades are categorized in ranges. (TIF) [file pone.0256543.s030.tif]

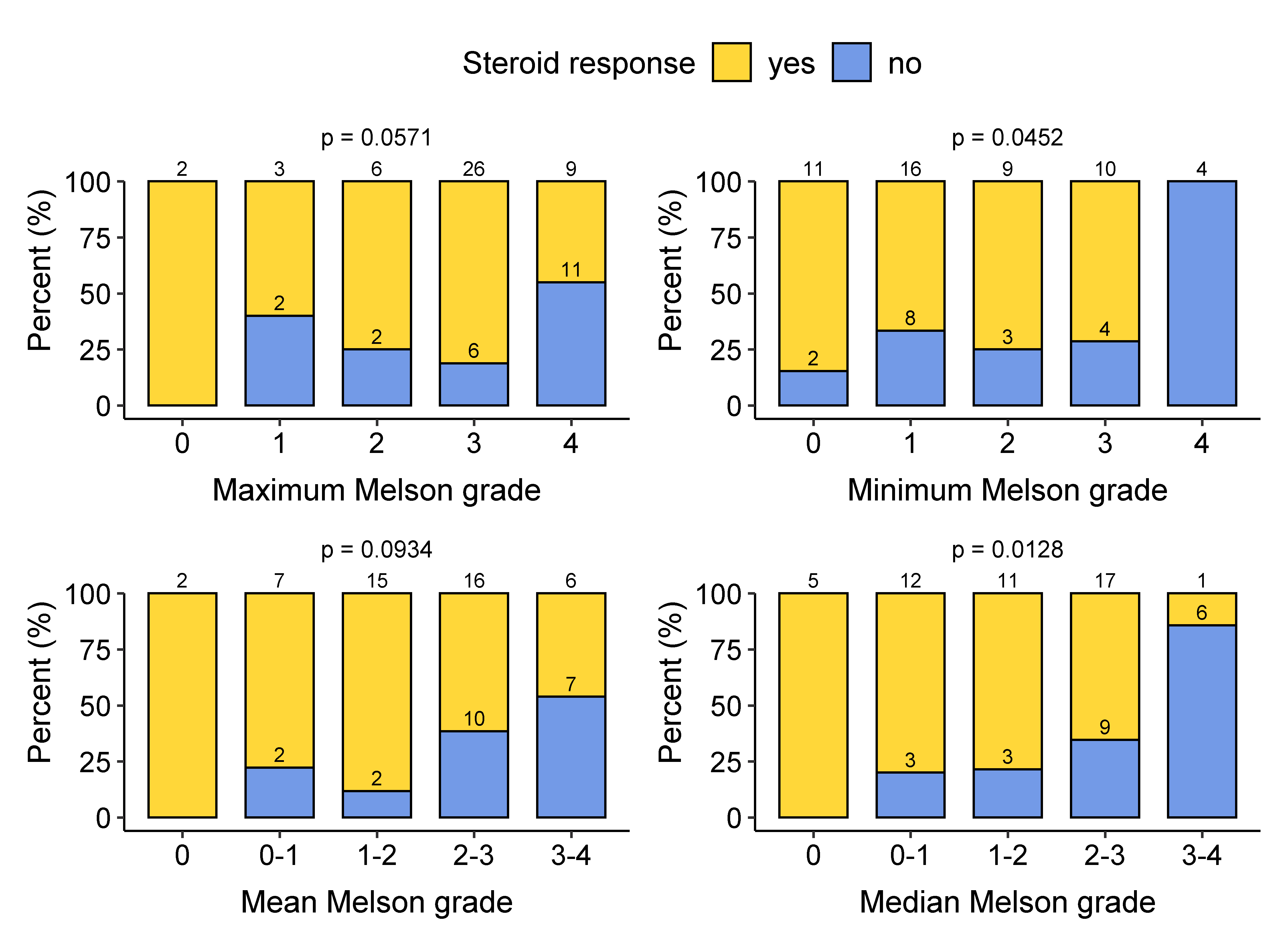

Supplement: S30 Fig — Graphical illustrations of contingency tables displaying case numbers and overall p-values of fisher´s exact count test. Mean and median grades are categorized in ranges. (TIF) [file pone.0256543.s031.tif]

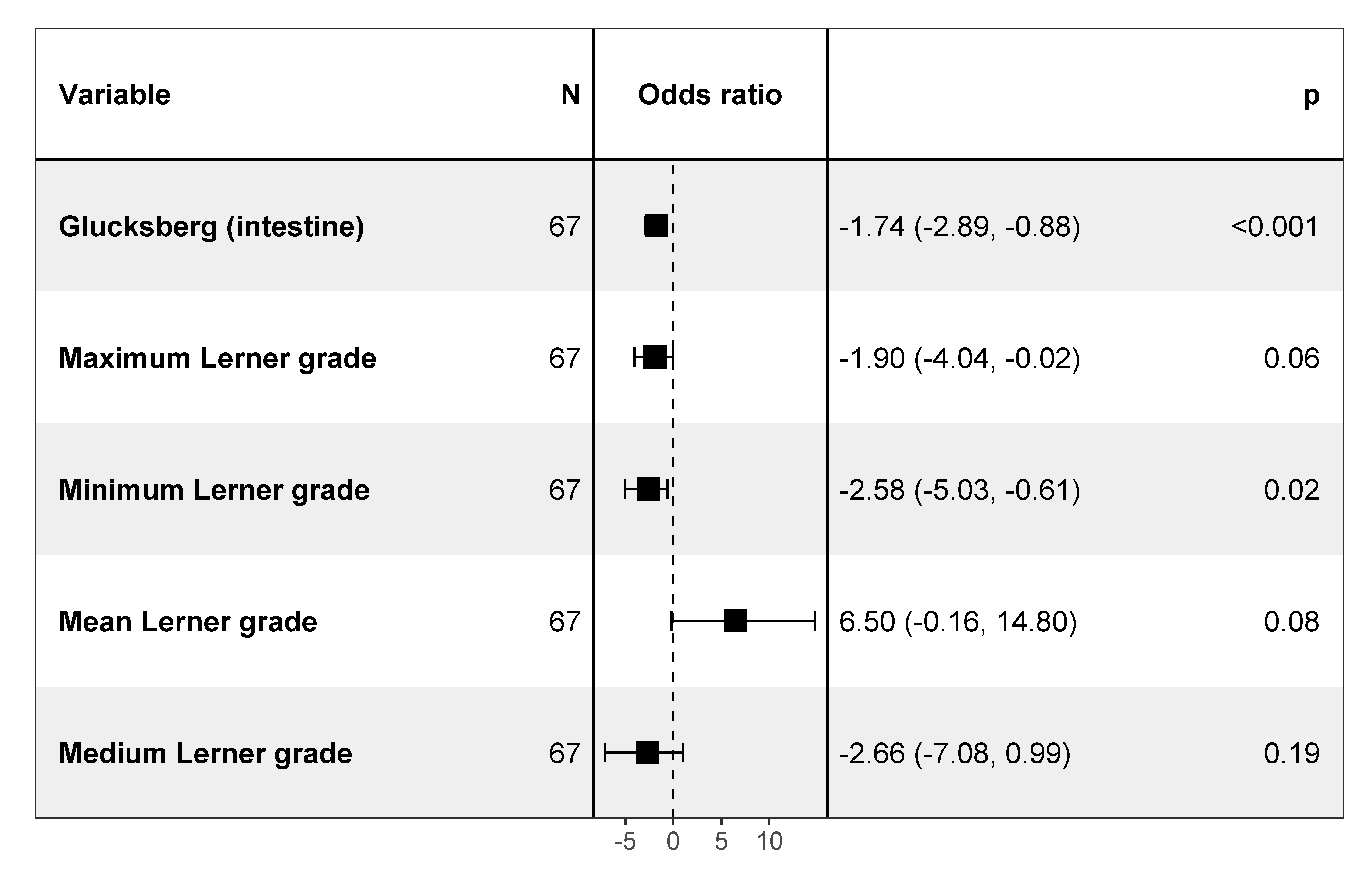

Supplement: S31 Fig — Forest plot of regression result depicting case numbers, odds ratios, confidence intervals and p-values. (TIF) [file pone.0256543.s032.tif]

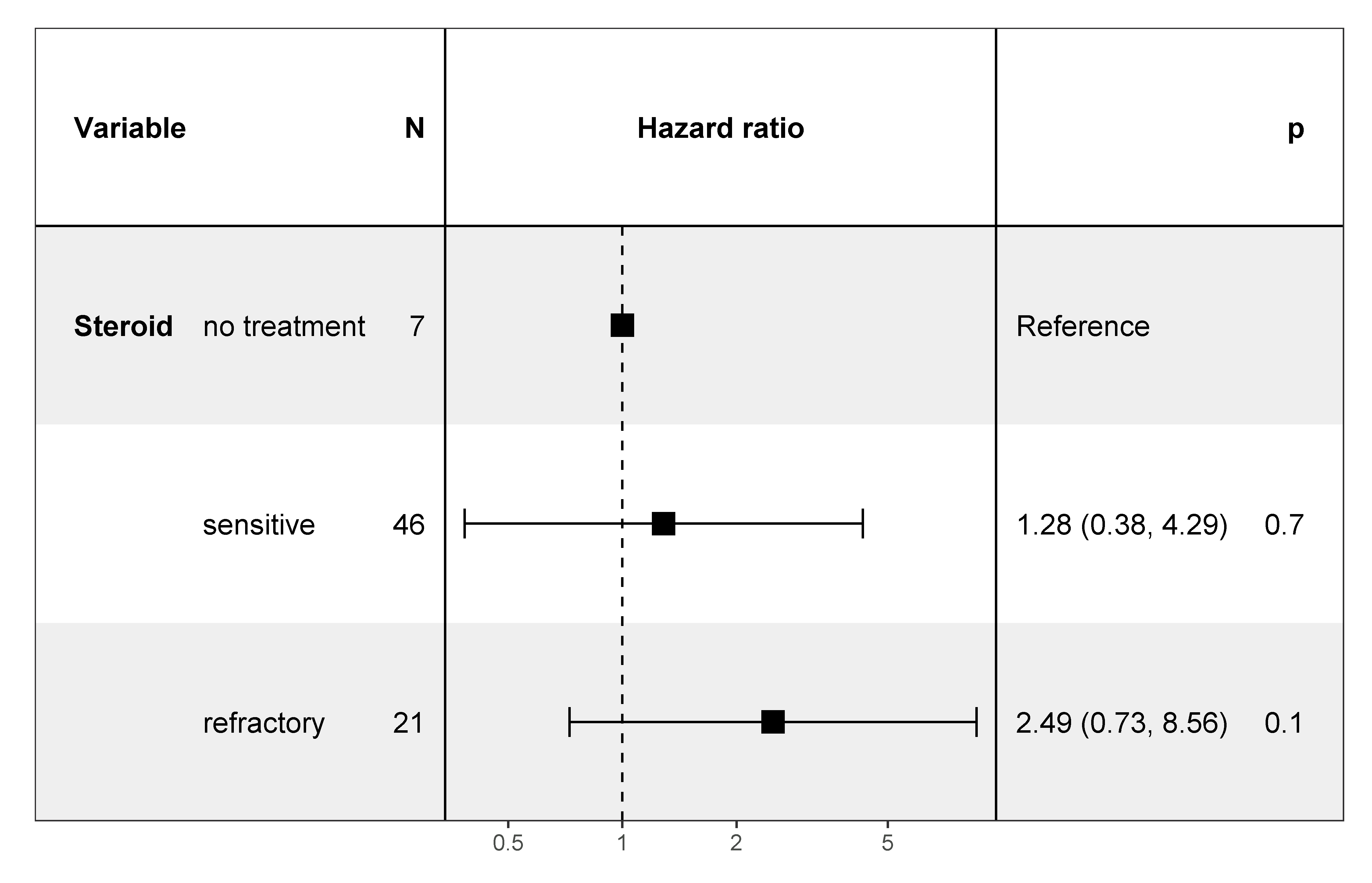

Supplement: S32 Fig — Forest plot of regression result depicting case numbers, hazard ratios, confidence intervals and p-values. (TIF) [file pone.0256543.s033.tif]

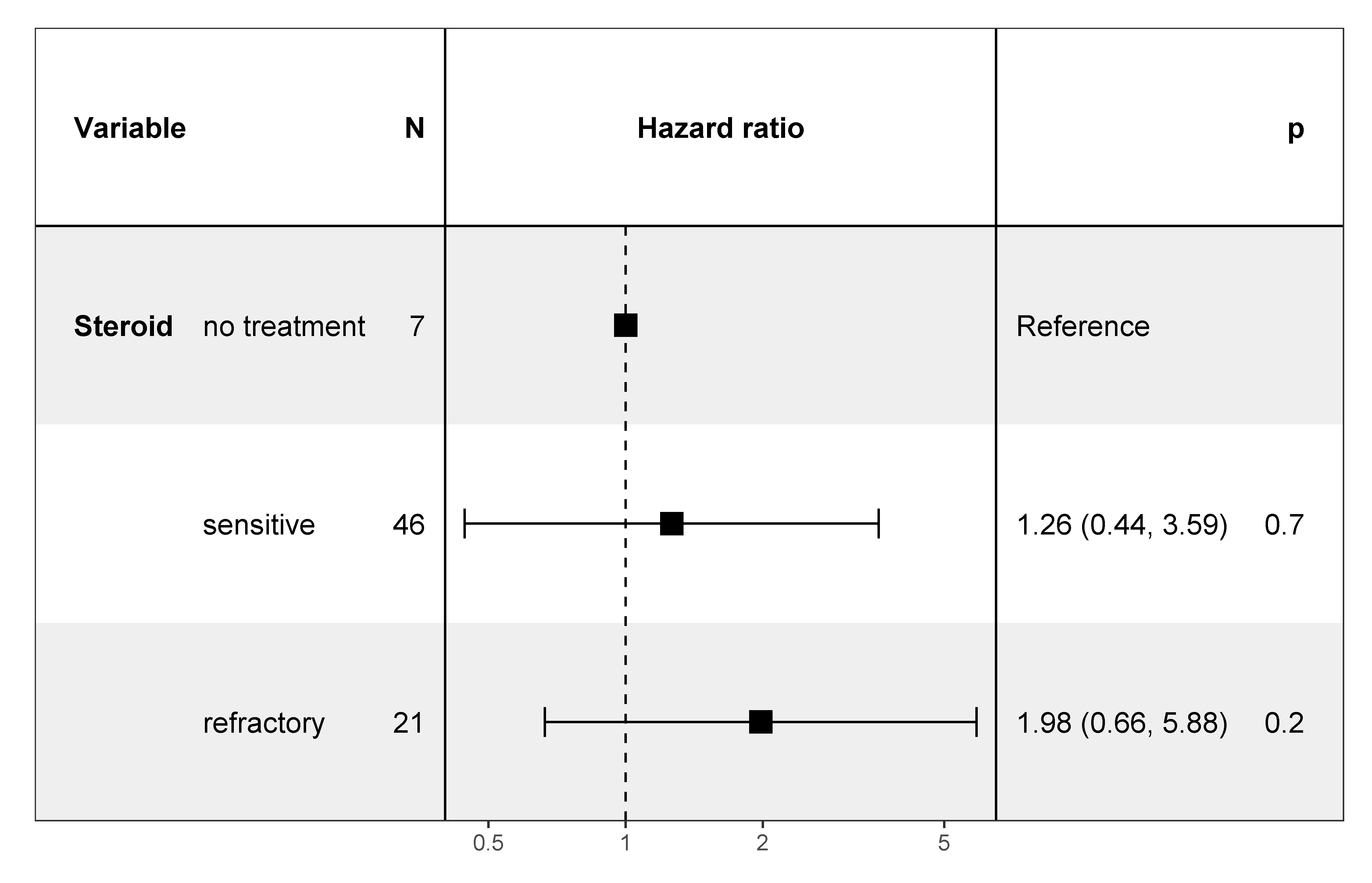

Supplement: S33 Fig — Forest plot of regression result depicting case numbers, hazard ratios, confidence intervals and p-values. (TIF) [file pone.0256543.s034.tif]

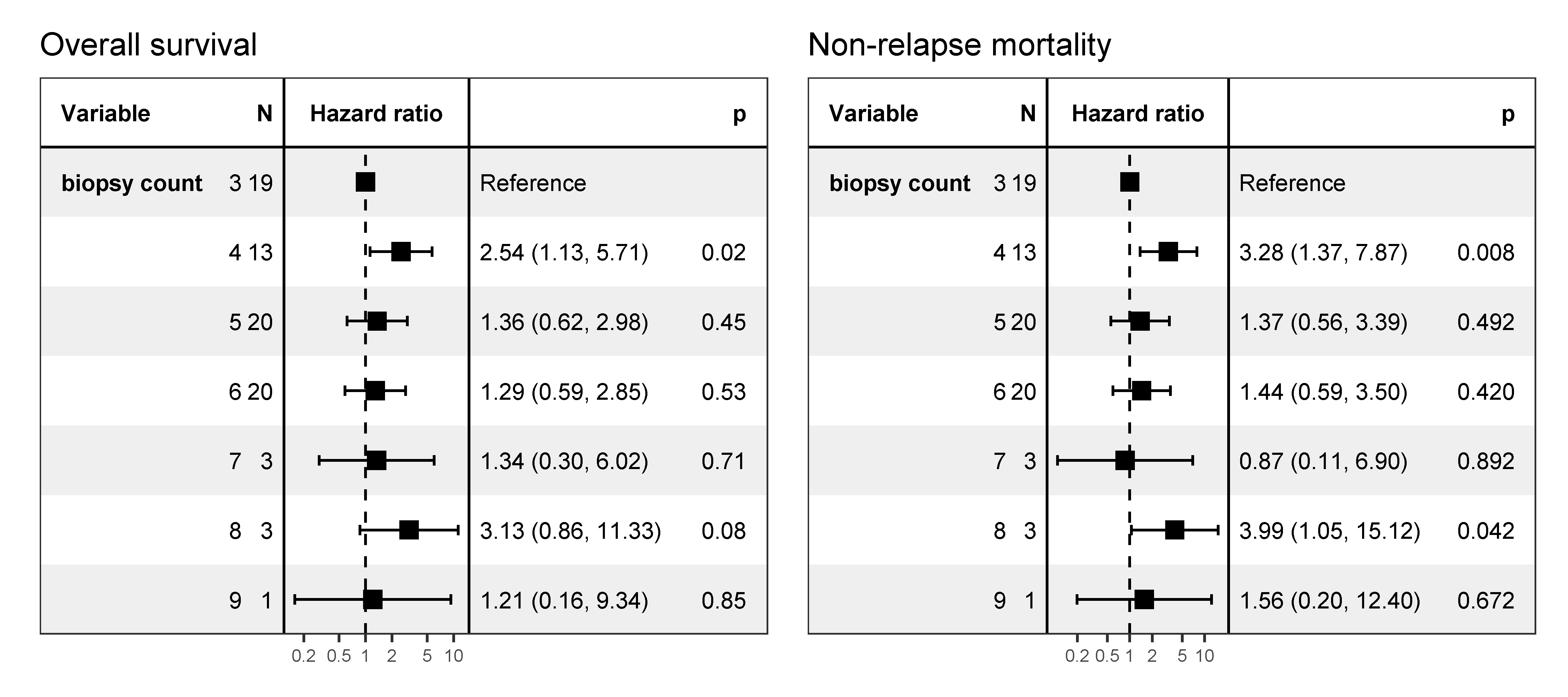

Supplement: S34 Fig — Forest plot of regression result depicting case numbers, hazard ratios, confidence intervals and p-values. (TIF) [file pone.0256543.s035.tif]
